# Cross-population amplitude coupling in high-dimensional oscillatory neural time series

**DOI:** 10.3389/fncom.2026.1703722

**Published:** 2026-02-03

**Authors:** Heejong Bong, Valérie Ventura, Eric A. Yttri, Matthew A. Smith, Robert E. Kass

**Affiliations:** 1 Department of Statistics, Purdue University, West Lafayette, IN, United States; 2 Department of Statistics and Data Sciences, Carnegie Mellon University, Pittsburgh, PA, United States; 3 Neuroscience Institute, Carnegie Mellon University, Pittsburgh, PA, United States; 4 Center for the Neural Basis of Cognition, Carnegie Mellon University, Pittsburgh, PA, United States; 5 Department of Biological Sciences, Carnegie Mellon University, Pittsburgh, PA, United States; 6 Department of Biomedical Engineering, Carnegie Mellon University, Pittsburgh, PA, United States; 7 Machine Learning Department, Carnegie Mellon University, Pittsburgh, PA, United States

**Keywords:** cross-region dynamic connectivity, Granger causality, high-dimensional time-series, latent factor models, local stationarity, multiset CCA

## Abstract

Neural oscillations have long been considered important markers of interaction across brain regions, yet identifying coordinated oscillatory activity from high-dimensional multiple-electrode recordings remains challenging. We sought to quantify time-varying covariation of oscillatory amplitudes across two brain regions, during a memory task, based on local field potentials recorded from 96 electrodes in each region. We extended Canonical Correlation Analysis (CCA) to multiple time series through the cross-correlation of latent time series. This, however, introduces a large number of possible lead-lag cross-correlations across the two regions. To manage that high dimensionality, we developed rigorous statistical procedures aimed at finding a small number of dominant lead-lag effects. The method correctly identified ground truth structure in realistic simulation-based settings. When we used it to analyze local field potentials recorded from the prefrontal cortex and visual area V4, we obtained highly plausible results. The new statistical methodology could also be applied to other slowly varying high-dimensional time series.

## 1 Introduction

**Figure 1:**
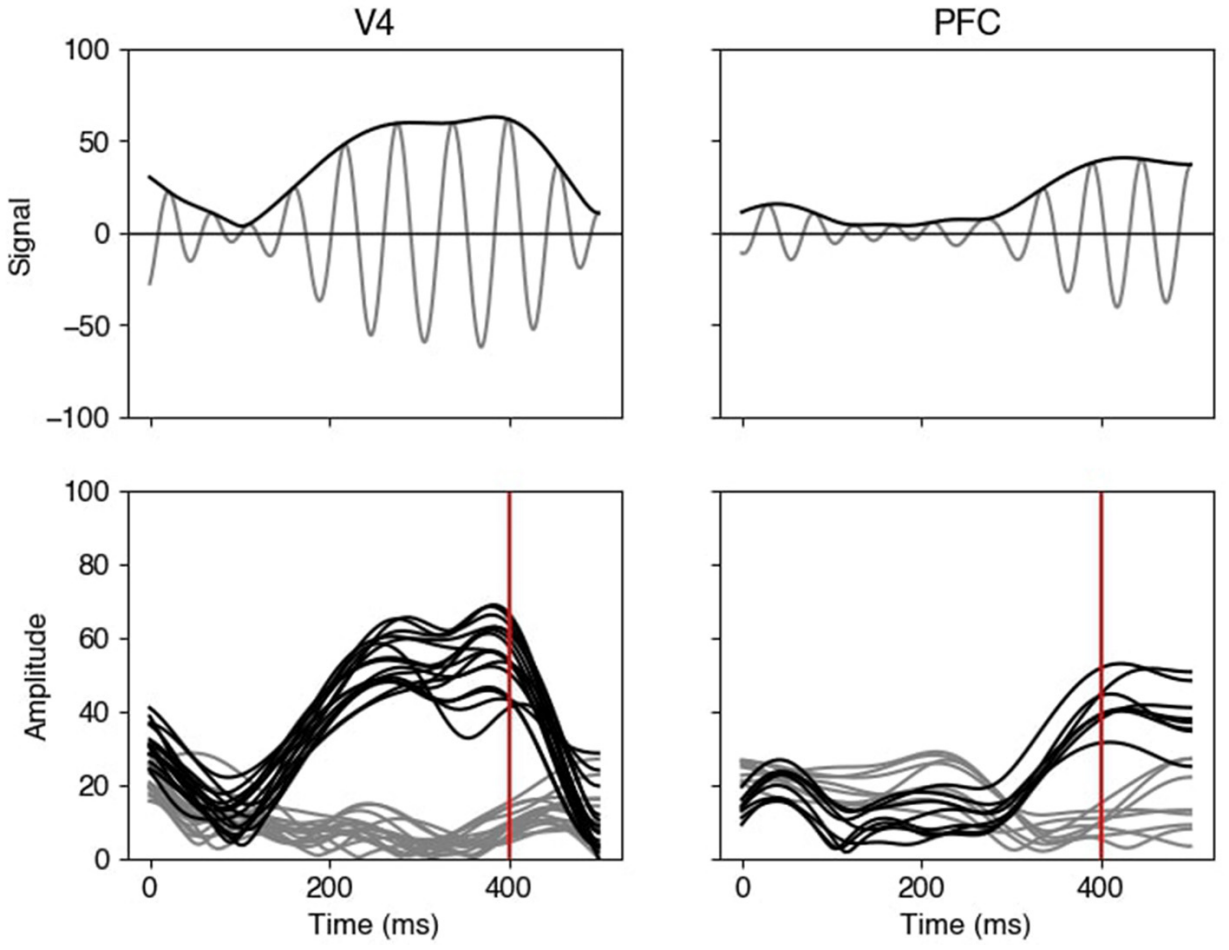
Bandpass-filtered LFP amplitude envelopes. The top panels display beta-range filtered LFPs (gray) together with their amplitude envelopes (black) from a pair of electrodes in V4 **(left)** and PFC **(right)** on a single trial, during the delay period of a working memory task. (The filtering is described in Section 3.2.1) The bottom panels display amplitude envelopes of active electrodes in the two brain regions for two trials, those for one trial in black and those for the other trial in gray. The beta amplitude envelopes show a consistent temporal pattern within each region, for both trials, and, at 400 ms (red vertical line), the two groups of amplitude envelopes from the two brain regions illustrate correlated behavior across the two trials in the sense that the black amplitude curves for the first trial are elevated, compared with the gray for the second trial, in both V4 and PFC. This correlation, however, varies across time.

Contemporary technologies for recording neural activity can produce multiple time series in each of two or more brain regions simultaneously (e.g., Jun et al., 2017; Steinmetz et al., 2018), enabling identification of cross-regional interactions relevant to cognitive processes that support behavior. One such process is working memory (Miller et al., 2018; Schmidt et al., 2019). During a variety of experimental working memory tasks, strong neural oscillations in the beta range (16–30 Hz) have been observed, leading to the proposal that beta oscillations serve to coordinate activity across regions (Miller et al., 2018). To identify coordinated oscillatory activity, many statistical methods based on coherence, or phase coupling, have been developed, studied, and applied (Belluscio et al., 2012; Klein et al., 2020; Ombao and Pinto, 2022; Urban et al., 2023). As shown in Figure 1, however, oscillatory amplitudes (amplitude envelopes) also vary substantially and may well exhibit statistical dependence across regions. Our aim is to detect whether these slowly-varying amplitude envelopes tend to fluctuate together, and to pinpoint when during a task such coupling occurs. This form of amplitude-based coordination reflects a different aspect of neural communication than phase-based measures and requires statistical tools that can accommodate smooth, non-oscillatory signal components. In this study, we develop methods for analyzing groups of slowly-varying multiple time series, and apply them to uncover coordinated amplitude activity between brain regions.

A common strategy for quantifying inter-areal association is to reduce multichannel recordings within each brain region to a small number of region-level summaries and then compute pairwise association measures between regions. For oscillatory neural signals, this is often done by computing amplitude envelope correlations after extracting instantaneous amplitudes via the Hilbert transform, based on region-level summaries (Bruns et al., 2000; Zamm et al., 2018; Raghavan et al., 2024). Constructing these summaries typically requires additional preprocessing, such as selecting a single representative channel per region (Bruns et al., 2000), applying spatial filters (Zamm et al., 2018), or designating channels based on expert judgment (Raghavan et al., 2024).

A similar dimension reduction principle underlies methods developed for non-oscillatory recordings such as fMRI. Time Delay (TD) analysis (Mitra et al., 2015, 2018; Raut et al., 2019) averages voxel-level signals within each region to obtain a single regional time-series. It then compares pairs of regional time series by measuring how much one signal is delayed relative to the other using cross-covariance, resulting in a single lag value for each region pair that can be further analyzed using PCA. Cyclicity analysis (Shahsavarani et al., 2020; Abraham et al., 2024) instead aggregates directionality measures across voxel-level interactions to produce a signed scalar measure that represents a region-level summary that captures region-level lead-lag relationships.

These approaches collapse multichannel activity into signals that mix task-relevant and task-irrelevant processes, which reduces sensitivity to between-region coupling and obscures the subpopulations that carry the interaction. We instead developed a method to learn low-dimensional and time-localized components directly from multichannel data, without spatial priors. By maximizing between-region dependence (relative to within-region variance), our approach increases sensitivity to inter-areal communication.

The data that motivated this work are local field potentials (LFPs; Buzsáki et al., 2012; Pesaran et al., 2018), recorded from prefrontal cortex (PFC) and visual area V4 during repeated trials of a working memory task (Khanna et al., 2020). PFC is generally considered to exert control aimed at areas involved in perceptual processing (Miller and Cohen, 2001), such as V4, which is active during the retention of higher-order visual information (e.g., color and shape) and attention to visual objects (Orban, 2008; Fries et al., 2001). Both regions exhibit strong oscillatory activity during visual working memory tasks, and their interaction, particularly through oscillatory coupling, has been implicated in supporting working memory function (Sarnthein et al., 1998; Liebe et al., 2012; Miller et al., 2018; Gregoriou et al., 2009).

**Figure 2:**
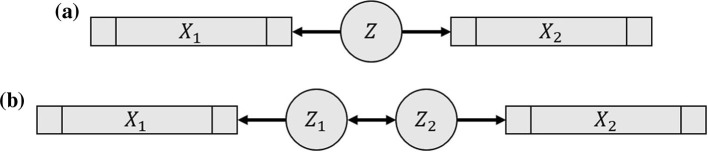
Graphical representation of pCCA models. **(a)** Model of Bach and Jordan (2005)), where *X*_1_ and *X*_2_ are random vectors and *Z* is a random variable. **(b)** A variation on **(a)** that facilitates the extension to the case when *X*_1_ and *X*_2_ are multivariate time series and (*Z*_1_, *Z*_2_) is a bivariate time series.

**Figure 3:**
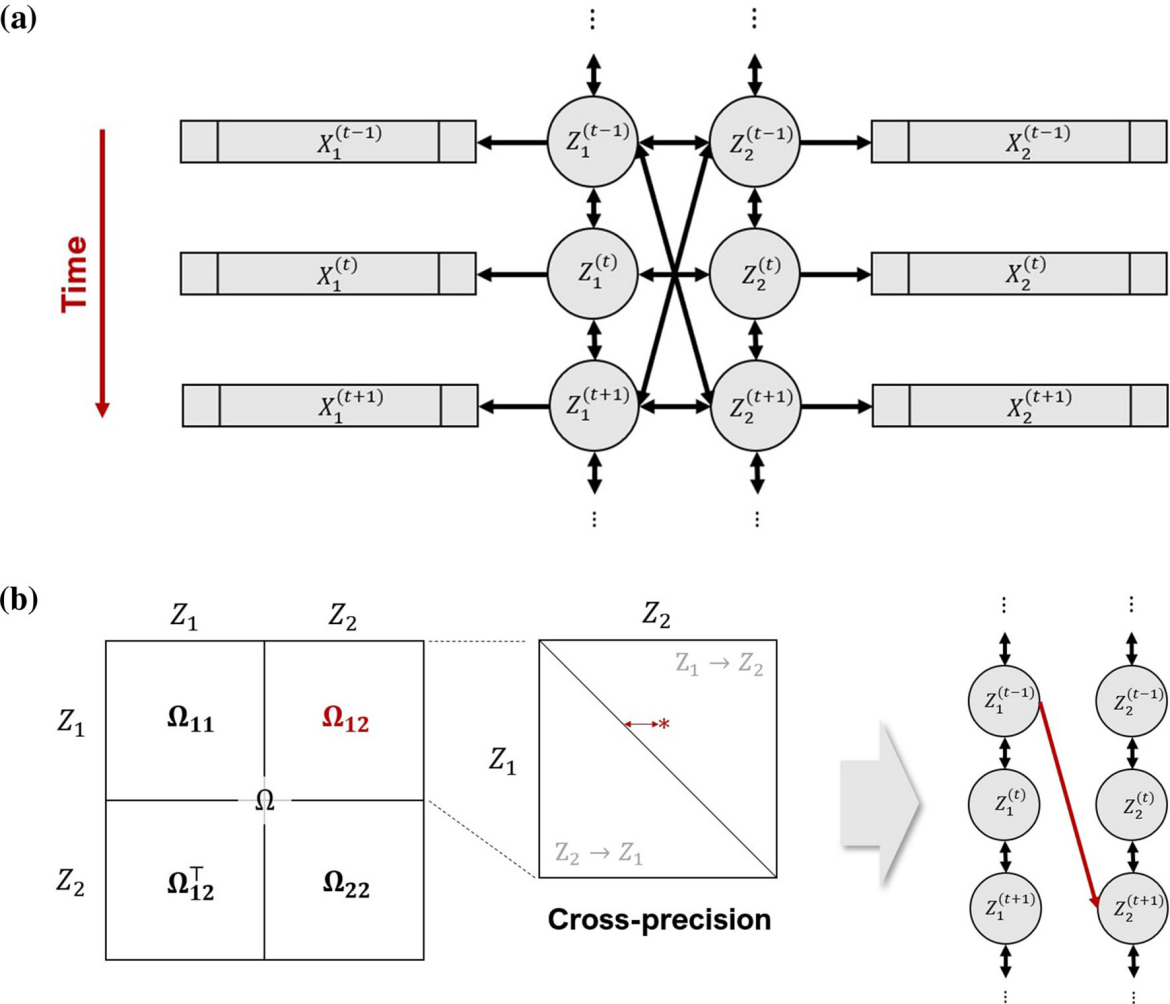
Extended pCCA model for two multivariate time series $X_{1}^{\left(t\right)}$ and $X_{2}^{\left(s\right)}$, *t, s* = 1, …, *T*. **(a)** Dynamic associations between vectors $X_{1}^{\left(t\right)}$ and $X_{2}^{\left(s\right)}$ are summarized by the dynamic associations between their associated 1-dimensional latent variables $Z_{1}^{\left(t\right)}$ and $Z_{2}^{\left(s\right)}$ represented by their cross-precision matrix Ω_12_. **(b)** When a significant cross-precision entry is identified, for example, the red star in the expanded view of Ω_12_, its coordinates and distance from the diagonal indicate at what time in the experiment connectivity between two brain areas occurs, and at what lead or lag. Here the red star is in the upper diagonal of Ω_12_, which means that, at this particular time, region 1 leads region 2, or *Z*_1_→*Z*_2_ in short (a non-zero entry in the lower diagonal would mean *Z*_2_→*Z*_1_). We represent this association by the red arrow on the right-most plot, with a lag of two units of time for illustration.

The data consist of 96 LFP time series for each brain region (192 time series in total), offering millisecond temporal precision, and are relatively precise spatially, as well. These signals reflect neural activity across large populations of neurons within each region and exhibit substantial spatial correlation. To summarize the cross-regional interactions more succinctly, dimensionality reduction becomes a natural and effective step (Gallego-Carracedo et al., 2022). If we were to analyze the data at a single time point, we would have repeated pairs of 96-dimensional observations, for which canonical correlation analysis (CCA) would be a standard tool to assess statistical dependence. Here we build on a latent variable formulation of CCA known as probabilistic CCA (pCCA), and extend it to handle temporally structured data, as illustrated in Figure 2b. In the extended model described in Section 2.2, each of the two high-dimensional time series is driven by a latent univariate time series, with the correlation between the two latent time series representing the statistical dependence we seek to identify (see Figure 3). Theorem 3.3 demonstrates that this model yields estimates equivalent to those from a generalization of classical CCA, called multiset CCA (Kettenring, 1971). This model-based framework allows for flexible modeling of covariance structure and facilitates principled high-dimensional inference.

Related latent variable methods include Gaussian Process Factor Analysis (GPFA; Lakshmanan et al., 2015; Semedo et al., 2020; Yu et al., 2009) and state-space models (Goris et al., 2014). GPFA assumes that latent time series follow a low-dimensional Gaussian process, while state-space models typically impose autoregressive dynamics. Although these methods were originally devised to capture shared low-dimensional structure within a single brain region, they can be embedded in a two-step approach to discover cross-regional interaction: (1) apply those methods separately to each brain region, and (2) compute cross-correlations between the resulting latent trajectories. While common in neuroscience, this strategy may lack statistical power when within-region activity does not align with cross-regional communication. For example, in recordings from macaque visual areas V1 and V2, Semedo et al. (2019)) found that communication occurred in a dedicated “communication subspace” distinct from the dominant within-region activity. Ignoring this distinction can lead to underestimating cross-regional coupling and reduced sensitivity (Semedo et al., 2020).

To address this, alternative methods have been proposed. Rodu et al. (2018)) extended a kernel version of CCA (KCCA; Hardoon et al., 2004) to develop *Dynamic Kernel CCA* (DKCCA), designed to detect time-varying connectivity between multivariate signals.

In this study, we introduce a more flexible alternative by leaving the latent covariance unspecified, allowing for a fully data-driven estimate of cross-regional correlations. This yields a partial correlation graph representation of neural interactions. Given the high dimensionality of the possible partial correlations across time lags, we adopt a sparse estimation framework (Friedman et al., 2008), resulting in our method: *La*tent *Dyn*amic analysis via *S*parse banded graphs (LaDynS). While we applied LaDynS to LFP recordings, it could also be used to analyze other slowly-varying multidimensional neural measurements made during repeated-trial cognitive tasks.

In Section 2, after defining the model and proving that it produces a time series generalization of CCA, we give details of the fitting procedure and discuss inference using clustered contiguous significant association based on a de-biased estimate of the precision matrix (Jankova et al., 2015) together with control of false discovery rate (Benjamini and Hochberg, 1995). We also use a state-space formulation to impose a local stationarity assumption (Ombao and Pinto, 2022), pointing out how this can produce a time-varying latent Granger causality assessment. Simulations in Section 3.2 show that our implementation of LaDynS is able to correctly identify the timing of interactions when applied to artificial data designed to be similar to those we analyzed, even in scenarios where existing methods exhibit low statistical power. The simulations support data-analytic results presented in Section 3.4, which show significant cross-regional interaction at roughly 400 ms after presentation of the stimulus. This timing coincides with the delay in visual cortical response seen after the onset of a visual stimulus in mice (Chen et al., 2022), humans (Del Cul et al., 2007; Yang et al., 2019), and macaques (Supèr et al., 2001; Schmolesky et al., 1998). The results reinforce the idea that PFC and V4 are involved, together, in working memory. We add a discussion in Section 4.

## 2 Methods

We begin by reviewing and reformulating probabilistic CCA (pCCA) in Section 2.1, which we then generalize to time series in Section 2.2 to yield a dynamic version of pCCA. We define the LaDynS model based on the log-likelihood function in Theorem 2.1. We go over the choice of regularization parameters in Section 2.3.1 and the fitting algorithm in Section 2.3.2. We discuss statistical inference in Section 2.4.

### 2.1 Probabilistic CCA for two random vectors

Given two random vectors $X_{1}\in {\mathbb{R}}^{d_{1}}$ and $X_{2}\in {\mathbb{R}}^{d_{2}}$, canonical correlation analysis (CCA; Hotelling, 1992) finds the sets of weights $w_{1}\in {\mathbb{R}}^{d_{1}}$ and $w_{2}\in {\mathbb{R}}^{d_{2}}$ that maximize Pearson's correlation between linear combinations $w_{1}^{⊤}X_{1}$ and $w_{2}^{⊤}X_{2}$. This can be rewritten as


$$\begin{array}{l} \sigma_{cc}=\max_{w_{k},k=1,2:w_{k}^{⊤}\Sigma_{kk}w_{k}=1}|w_{1}^{⊤}\Sigma_{12}w_{2}|, \end{array}$$ (1)

where Σ_*kk*_ = Var(*X*_*k*_) is the covariance matrix of *X*_*k*_, *k* = 1, 2, and Σ_12_ = Cov(*X*_1_, *X*_2_) the cross-covariance matrix between *X*_1_ and *X*_2_. The sample estimator ${\hat{\sigma }}_{cc}$ is obtained by replacing Σ_*kk*_ and Σ_12_ with their sample analogs ${\bar{\Sigma }}_{kk}$ and ${\bar{\Sigma }}_{12}$ respectively. The maximizing weights ŵ_*k*_ and linear combinations ${Ẑ}_{k}={ŵ}_{k}^{⊤}X_{k}$ are referred to as the canonical weights and canonical variables, respectively.

Probabilistic CCA assumes that *X*_1_ and *X*_2_ are driven by a common one dimensional latent variable *Z*:


$$\begin{array}{l} \begin{array}{l} X_{k}|Z=\mu_{k}+Z\cdot \beta_{k}+\epsilon_{k},\;k=1,2,\\ \;Z\sim N\left(0,1\right), \end{array} \end{array}$$ (2)

where $\mu_{k}\in {\mathbb{R}}^{d_{k}}$ and $\beta_{k}\in {\mathbb{R}}^{d_{k}}$ are mean vectors and factor loadings, respectively, and $\epsilon_{k}\overset{\text{indep}}{\sim }\mathrm{MVN}\left(0,\Phi_{k}\right)$ (Bach and Jordan, 2005). Figure 2a depicts the dependence of *X*_1_ and *X*_2_ on *Z*. The parameters in Equations 1, 2 both yield the same estimate of σ_*cc*_; see Theorem 3.1 for more details.

Next, we introduce an alternative pCCA extension that assigns distinct latent variables for *X*_1_ and *X*_2_, as depicted in Figure 2b. Specifically, we assume that


$$\begin{array}{l} X_{k}|Z_{k}=\mu_{k}+Z_{k}\cdot \beta_{k}+\epsilon_{k} \end{array}$$ (3)

where $\mu_{k}\in {\mathbb{R}}^{d_{k}}$, $\beta_{k}\in {\mathbb{R}}^{d_{k}}$ and $\epsilon_{k}\overset{\text{indep}}{\sim }\mathrm{MVN}\left(0,\Phi_{k}\right)$ are defined as in Equation 2, and (*Z*_1_, *Z*_2_) are bivariate normally distributed:


$$\begin{array}{l} \left(\begin{array}{c} Z_{1}\\ Z_{2} \end{array}\right)\sim \mathrm{MVN}\left(\left(\begin{array}{c} 0\\ 0 \end{array}\right),\left(\begin{array}{cc} 1 & \sigma_{12}\\ \sigma_{12} & 1 \end{array}\right)\right). \end{array}$$ (4)

Like the original method, the alternative pCCA yields the same estimate of σ_*cc*_. We prove this equivalence in Theorem 3.2.

### 2.2 Probabilistic CCA for two time series of random vectors

Suppose now that we are interested in the correlation dynamics between two times series of random vectors $X_{1}^{\left(t\right)}\in {\mathbb{R}}^{d_{1}}$ and $X_{2}^{\left(t\right)}\in {\mathbb{R}}^{d_{2}}$, *t* = 1, 2, …, *T*. For each time *t*, we use Equation 3 to model the dependence of $X_{k}^{\left(t\right)}$ on its associated latent variable $Z_{k}^{\left(t\right)}$:


$$\begin{array}{l} X_{k}^{\left(t\right)}|Z_{k}^{\left(t\right)}=\mu_{k}^{\left(t\right)}+\beta_{k}^{\left(t\right)}\cdot Z_{k}^{\left(t\right)}+\epsilon_{k}^{\left(t\right)},\;k=1,2, \end{array}$$ (5)

where $\mu_{k}^{\left(t\right)}$, $\beta_{k}^{\left(t\right)}$ and $\epsilon_{k}^{\left(t\right)}\overset{\text{indep}}{\sim }\mathrm{MVN}\left(0,\Phi_{k}^{\left(t\right)}\right)$ are defined as in Equation 3.

Then for each *t* we could define a parameter $\sigma_{12}^{\left(t\right)}$ as in Equation 4 to capture population-level association between $X_{1}^{\left(t\right)}$ and $X_{2}^{\left(t\right)}$ at *t*. But because we are also interested in lagged associations between $X_{1}^{\left(t\right)}$ and $X_{2}^{\left(s\right)}$ for *s*≠*t*, we replace the bivariate Equation 4 for $Z_{1}^{\left(t\right)}$ and $Z_{2}^{\left(t\right)}$ for a given *t* by a global model for all *t* = 1, …, *T*:


$$\begin{array}{l} {\left({\left(Z_{1}^{\left(t\right)}\right)}_{t=1,\ldots ,T},{\left(Z_{2}^{\left(t\right)}\right)}_{t=1,\ldots ,T}\right)}^{⊤}\sim \mathrm{MVN}\left(0,\Sigma \right),\;\mathrm{diag}\left(\Sigma \right)=\text{1}, \end{array}$$ (6)

where Σ captures all simultaneous and lagged associations within and between the two time series jointly. Equation 3a illustrates the dependence structure of this model. to highlight the auto-correlations Σ_*kk*_, *k* = 1, 2, within and cross-correlations Σ_12_ between the times series, and denote each element of Σ_*ij*_ by Σ_*ij*_(*t, s*), (*t, s*)∈[*T*]^2^. Then Σ_12_(*t, t*) has the same interpretation as σ_12_ in EqXXXX and when *t*≠*s*, Σ_12_(*t, s*) measures the lead-lag association between the time series at latency |*t*−*s*|. We decompose Σ and its inverse Ω as


$$\Sigma =(\begin{array}{cc} \Sigma_{11} & \Sigma_{12}\\ \Sigma_{12}^{⊤} & \Sigma_{22} \end{array})\Omega =(\begin{array}{cc} \Omega_{11} & \Omega_{12}\\ \Omega_{12}^{⊤} & \Omega_{22} \end{array})$$ (7)

to highlight the auto-correlations Σ_11_ and Σ_22_ within and cross-correlations Σ_12_ between the time series, and denote by $\Sigma_{kl}^{\left(t,s\right)}$, (*t, s*)∈[*T*]^2^, the elements of Σ_*kl*_. Then $\Sigma_{12}^{\left(t,t\right)}$ for some fixed *t* has the same interpretation as σ_12_ in Equation 2. Further, $-\Omega_{12}^{\left(t,s\right)}/\sqrt{\Omega_{11}^{\left(t,t\right)}\Omega_{22}^{\left(s,s\right)}}$ is the partial correlation between the two latent time series at times *t* and *s*. Thus, when an element of Ω_12_ is non-null, depicted by the red star in the expanded display in Figure 3b, its coordinates (*t, s*) and distance (*t*−*s*) from the diagonal indicate at what time in the trial a connectivity happens between two time series, and at what lead or lag, respectively. In our neuroscience application, they represent the timing of connections and the direction of information flow between two brain regions. As is common with latent variable models, the signs of the latent covariance Σ and therefore the latent precision Ω are not identifiable. This non-identifiability is benign for inference on the directionality in lead-lag connectivity, which is encoded in the support of Ω_12_, rather than by their signs (see Figure 3b). The directionality remains interpretable even though the signs of the partial correlations are unidentifiable. Therefore, in the following sections, we focus on the estimation and inference for the magnitude of associations, rather than their sign.

As the equivalence between a non-distributional method (CCA) and its probabilistic representation (pCCA), there exists a similar connection between the multiset generalization of CCA introduced by Kettenring (1971)) and the dynamic pCCA model in Equations 5, 6. Multiset CCA applied to 2*T* random vectors $\left\{X_{1}^{\left(t\right)},X_{2}^{\left(t\right)}:t=1,\ldots ,T\right\}$ finds weights $\left\{w_{1}^{\left(t\right)},w_{2}^{\left(t\right)}:t=1,\ldots ,T\right\}$ that maximize a notion of correlation among linear combinations $\left\{w_{1}^{\left(t\right)⊤}X_{1}^{\left(t\right)},w_{2}^{\left(t\right)⊤}X_{2}^{\left(t\right)}:t=1,\ldots ,T\right\}$. In particular, the GENVAR extension minimizes the generalized variance of these linear combinations, defined as the determinant of their correlation matrix (Wilks, 1932), which we refer to as the canonical correlation matrix:


$$\begin{array}{l} {\hat{w}}_{1}^{\left(1\right)},\ldots ,{\hat{w}}_{2}^{\left(T\right)}=\arg\min_{w_{1}^{\left(1\right)},\ldots ,w_{2}^{\left(T\right)}}\det(\bar{\text{Var}}[{\left(w_{1}^{(t)⊤}X_{1}^{\left(t\right)}\right)}_{t=1,\ldots ,T},\\ {\left(w_{2}^{(t)⊤}X_{2}^{\left(t\right)}\right)}_{t=1,\ldots ,T}]) \end{array}$$ (8)

where $\bar{\mathrm{Var}}$ denotes the sample variance-covariance matrix and the weights $w_{k}^{\left(t\right)}$ are scaled so that every diagonal entry of the matrix is 1. In Theorem 3.3 we show that the maximum likelihood estimators from the dynamic pCCA model recover the same canonical weights $\left\{w_{1}^{\left(1\right)},\ldots ,w_{2}^{\left(T\right)}\right\}$ and the canonical correlation matrix $\hat{\Sigma }$ as the GENVAR formulation of multiset CCA. Under this equivalence, the MLEs minimize


$$\begin{array}{l} \log\det\left(\Sigma \right)+\mathrm{tr}\left(\Sigma^{-1}\bar{\Sigma }\right), \end{array}$$ (9)

An objective that motivates the Latent Dynamic Analysis via Sparse Banded Graphs (LaDynS) developed in the next section. The objective above does not involve any component of the observation model Equation 5; thus, the estimators do not depend on a Gaussian assumption for $X_{k}^{\left(t\right)}|Z_{k}^{\left(t\right)}$. They only require approximate normality of the latent factors $Z_{k}^{\left(t\right)}$, which is often plausible since each $Z_{k}^{\left(t\right)}$ is a weighted sum of many coordinates of $X_{k}^{\left(t\right)}$.

### 2.3 Latent dynamic analysis via sparse banded graphs (LaDynS)

**Figure 4:**
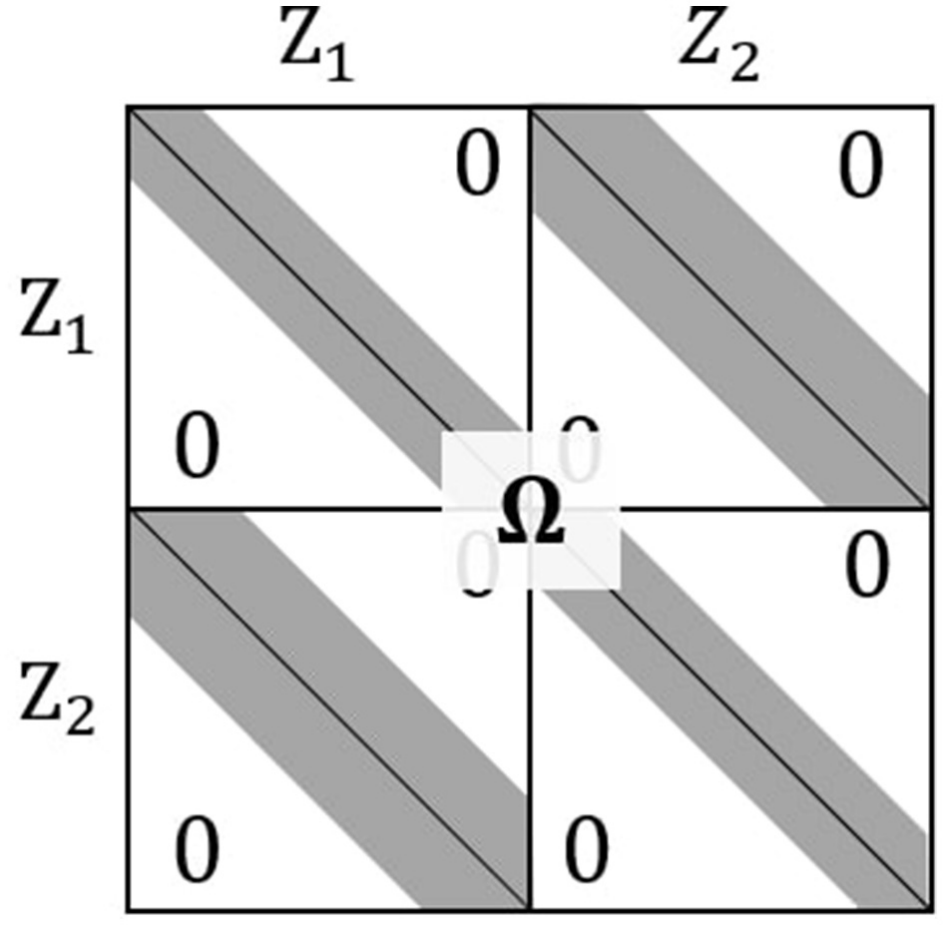
The elements of Ω_*kk*_, *k* = 1, 2, and Ω_12_ are set to zero outside of the gray bands of widths (1+2*d*_auto_) and (1+2*d*_cross_), respectively.

Our goal is to estimate the association dynamics between two multivariate time series of length *T* using the covariance matrix Σ of their associated latent time series in Equation 6. However, the prohibitive number of parameters in Σ means its estimation is prone to errors, especially when *T* is large. We reduce their number by regularizing Ω = Σ^−1^, rewriting logdet(Σ) = logdet(Ω^−1^) = −logdet(Ω), and assuming that Ω has the banded structure depicted in Figure 4.

***Definition* 0.0.1 (LaDynS).** Given *N* simultaneously recorded pairs of multivariate time series {_*X*_1[*n*]_, *X*_2[*n*]_}*n* = 1, …, *N*_, and a 2*T*×2*T* sparsity matrix Λ with element $\Lambda_{kl}^{\left(t,s\right)}$ regularizing $\left|\Omega_{kl}^{\left(t,s\right)}\right|$, *k, l* = 1, 2, LaDynS finds weights $\left\{{ŵ}_{k}^{\left(t\right)},t=1,2,\ldots ,T,k=1,2\right\}$ and precision matrix $\hat{\Omega }$ that minimize the penalized negative log-likelihood:


$$\begin{array}{l} -\log\det\left(\Omega \right)+\mathrm{tr}\left(\Omega \bar{\Sigma }\right)+||\Lambda ⊙\Omega |{|}_{1}, \end{array}$$ (10)

where $\bar{\Sigma }=\bar{\mathrm{Var}}\left[w_{1}^{\left(1\right)⊤}X_{1}^{\left(1\right)},\ldots ,w_{2}^{\left(T\right)⊤}X_{2}^{\left(T\right)}\right]$ satisfies $\mathrm{diag}\left(\bar{\Sigma }\right)=\text{1}$, ⊙ denotes the Hadamard product operator such that (*A*⊙*B*)_*ij*_ = *A*_*ij*_×*B*_*ij*_, $\left||A|{|}_{1}=\underset{i,j}{\sum }|A_{ij}\right|$, and


$$\Lambda_{kl}^{\left(t,s\right)}=\{\begin{array}{ll} \lambda_{\text{cross}}, & k\neq l\;\;\text{and}\;\;0<\;|t-s|\leq d_{\text{cross}},\\ \lambda_{\text{auto}}, & k=l\;\;\text{and}\;\;0<\;|t-s|\leq d_{\text{auto}},\\ \lambda_{\text{diag}}, & t=s,\\ \infty , & \text{otherwise}, \end{array}$$

which constrains auto-precision and cross-precision elements within a specified range.

In our neuroscience application, in particular, it is reasonable to assume that lead-lag relationships occur with delay less than temporal bandwidth *d*_cross_, which can be determined by the maximal transmission time in synaptic connections between two brain regions under study. We thus set $\Lambda_{12}^{\left(t,s\right)}=\infty$ when |*t*−*s*|>*d*_cross_ to force the corresponding cross-precision elements to zero and thus impose a banded structure on Ω_12_. We apply sparsity constraint $\Lambda_{12}^{\left(t,s\right)}=\lambda_{\text{cross}}>0$ on the remaining off-diagonals of Ω_12_ to focus our discovery of sparse dominant associations and reduce the effective parameter size. We proceed similarly with the auto-precision matrices Ω_11_ and Ω_22_, using penalty λ_auto_ and temporal bandwidth *d*_auto_. Unless domain knowledge is available, we recommend that *d*_auto_ be set to the largest significant auto-correlation across all observed time series $X_{k,i}^{\left(t\right)}$, *k* = 1, 2, *i* = 1, …, *N*, and impose no further sparsity (λ_auto_ = 0) unless there is reason to expect it.

Notice that, for simplicity, we grouped the elements of Λ into diagonal and off-diagonal elements and assigned the same penalties, λ_cross_, λ_auto_ = 0, and λ_diag_, within each group.

Again, LaDynS estimates the magnitude of the associations, but their signs remain non-identifiable. Accordingly, our results in Section 3 report and display the absolute values of the estimated variance and precision elements.

#### 2.3.1 Choosing regularization parameters

In graphical LASSO (gLASSO) problems, where the aim is to recover correct partial correlation graphs, penalties are often chosen to minimize the predictive risk (Shao, 1993; Zou et al., 2007; Tibshirani et al., 2012). Our aim is different: only the partial cross-precision matrix Ω_12_ is of substantive interest, and because minimizing the predictive risk does not select models consistently (Shao, 1993; Zhu and Cribben, 2018) and may thus fail to retrieve non-zero elements of Ω_12_, we choose instead a value of λ_cross_ that controls the number of false cross-precision discoveries. We proceed by permuting the observed time series in one brain region to create a synthetic dataset that contains no cross-region correlation, then applying LaDynS to that data for a range of values of λ_cross_ and recording the resulting number of significant partial correlation estimates, which are necessarily spurious. We use the smallest λ_cross_ that yields fewer false discoveries than a chosen threshold. We expect this regularization to make similarly few false discoveries on experimental data.

Finally, if $\hat{\Sigma }$ cannot be inverted, as is the case for the band-pass filtered experimental data we analyze in Section 3, we penalize its diagonal by λ_diag_>0. We explain the specific calibration We used the analyzed datasets and studied the properties in Section 3.2.2.

#### 2.3.2 Fitting LaDynS using coordinate descent

Equation 10 is not a convex function of the weights and precision elements (although it is not impossible that it may be for some particular Σ), and its convex relaxation is unknown, so it is difficult to find its global minimum. The coordinate descent algorithm described below finds a minimum, possibly local, since it may be sensitive to the choice of initial parameter values. However, the simulation in Supplementary Section S2.2 suggests that the algorithm's sensitivity to initial values has little impact on its ability to recover the correct connectivity.

Assuming that all canonical weights $w_{k}^{\left(t\right)}$ are fixed, Equation 10 reduces to the gLASSO problem:


$$\begin{array}{l} \underset{\Omega }{\mathrm{argmin}}-\log\det\left(\Omega \right)+\mathrm{tr}\left(\Omega \bar{\Sigma }\right)+||\Lambda ⊙\Omega |{|}_{1}, \end{array}$$ (11)

which we can solve efficiently using a number of existing algorithms; here we use the P-gLASSO algorithm of Mazumder and Hastie (2012)). Then, assuming that all parameters are fixed but a single weight $w_{k}^{\left(t\right)}$, Equation 10 can be rearranged as the linear problem:


$$\begin{array}{l} \underset{w_{k}^{\left(t\right)}}{\mathrm{argmin}}\sum_{\left(l,s\right)\neq \left(k,t\right)}w_{k}^{\left(t\right)⊤}\bar{\mathrm{Cov}}\left[X_{k}^{\left(t\right)},X_{l}^{\left(s\right)}\right]w_{l}^{\left(s\right)}\Omega_{kl}^{\left(t,s\right)} \end{array}$$ (12)

$$\begin{array}{l} \text{ s.t. }w_{k}^{\left(t\right)⊤}\bar{\mathrm{Var}}\left[X_{k}^{\left(t\right)}\right]w_{k}^{\left(t\right)}=1, \end{array}$$

for which an analytical solution is available. That is, our algorithm alternates between updating Ω and the weights $w_{k}^{\left(t\right)}$ until the objective function in Equation 10 converges. Its computational cost is inexpensive: a single iteration on our cluster server (with 11 Intel(R) Xeon(R) CPU 2.90 GHz processors) took, on average, less than 0.8 s, applied to the experimental data in Section 3.4. A single fit on the same data took 47 iterations for around 33.57 s until the objective function converged at threshold 0.001.

Algorithm 1 presents a high-level pseudocode sketch of the coordinate descent algorithm. See Supplementary Section S2.1 for details and Python package ladyns on github.com/HeejongBong/ladyns.

Algorithm 1 Coordinate descent algorithm to fit LaDynS. 
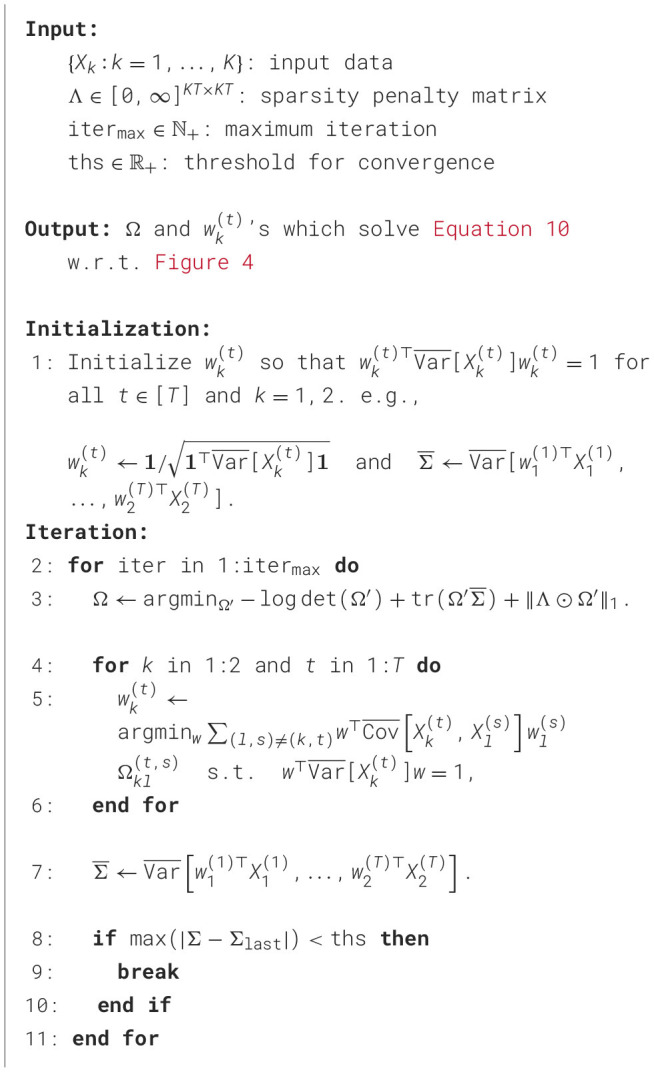

### 2.4 Inference for associations between two vector time series

Let $\hat{\Omega }$ and ${ŵ}_{k}^{\left(t\right)}$, *t* = 1, …, *T*, *k* = 1, 2, be the LaDynS estimates of canonical precision matrix and canonical weights, and $\bar{\Sigma }=\bar{\mathrm{Var}}\left[{ŵ}_{1}^{\left(1\right)⊤}X_{1}^{\left(1\right)},\ldots ,{ŵ}_{2}^{\left(T\right)⊤}X_{2}^{\left(T\right)}\right]$ be the empirical covariance of the estimated latent variables, defined in Equation 10. Note that $\hat{\Omega }\neq {\bar{\Sigma }}^{-1}$ since $\hat{\Omega }$ is constrained to be sparse. Based on these estimates, we want to identify the non-zero partial cross-correlations in Ω_12_, which correspond to the epochs of association between the two time series.

Formal inference methods for Ω based on its LaDynS estimate (Equation 10) are not available, but because LaDynS reduces to graphical LASSO (gLASSO) when the weights $w_{k}^{\left(t\right)}$ in Equation 11 are fixed, we co-opt gLASSO inference methods. Specifically, Jankova et al. (2015)) suggested de-sparsifying the gLASSO estimate $\hat{\Omega }$ according to


$$\begin{array}{l} \tilde{\Omega }=2\hat{\Omega }-\hat{\Omega }\left(\bar{\Sigma }+\lambda_{\text{diag}}I_{T}\right)\hat{\Omega }, \end{array}$$ (13)

and proved that, under mild assumptions and as *n* → ∞, each entry of $\tilde{\Omega }$ satisfies the Central Limit Theorem with center the true precision Ω:


$$\begin{array}{l} \forall \left(t,s\right),\;\frac{\left({\tilde{\Omega }}_{12}^{\left(t,s\right)}-\Omega_{12}^{\left(t,s\right)}\right)}{\sqrt{\mathrm{Var}\left[{\tilde{\Omega }}_{12}^{\left(t,s\right)}\right]}}\overset{d}{\to }N\left(0,1\right). \end{array}$$ (14)

The assumptions include independence across experimental trials, irrepresentability condition, and bounded eigenvalues of Ω [see Assumptions (A1) and (A2) in Jankova et al. (2015))]. While some of these conditions may be strong and difficult to verify in practice, they are commonly imposed in the literature on sparse graph estimation with ℓ_1_ regularization. In Supplementary Section S3.2, we examine the asymptotic normality of the de-sparsified LaDynS estimate using simulation.

We applied this result to the de-sparsified LaDynS estimate of Ω, even though we do not quite have a gLASSO setup, and we verified by simulation that its elements are indeed approximately normal in Section 3.2.2. Jankova et al. (2015)) also proposed an estimator of $\mathrm{Var}\left[{\tilde{\Omega }}_{12}^{\left(t,s\right)}\right]$ that is likely to be downward-biased in our framework since estimating the canonical weights $w_{k}^{\left(t\right)}$ induces extra randomness. Instead, we use the bootstrap estimate $\hat{\mathrm{Var}}\left[{\tilde{\Omega }}_{12}^{\left(t,s\right)}\right]$ described at the end of this section. We then rely on Equation 14 to obtain *p*-values


$$\begin{array}{l} p^{\left(t,s\right)}=2-2\Phi \left(|{\tilde{\Omega }}_{12}^{\left(t,s\right)}|/\sqrt{\hat{\mathrm{Var}}\left[{\tilde{\Omega }}_{12}^{\left(t,s\right)}\right]}\right) \end{array}$$ (15)

to test $H_{0}^{\left(t,s\right)}:\Omega_{12}^{\left(t,s\right)}=0$, for each (*t, s*)∈[*T*]^2^ within *d*_cross_ of the diagonal of Ω_12_.

*Permutation bootstrap estimate of* $\;\mathrm{Var}\left[{\tilde{\Omega }}_{12}^{\left(t,s\right)}\right]$*:* a permutation bootstrap sample ${\left\{X_{1\left[n\right]}^{*},X_{2\left[n\right]}^{*}\right\}}_{n=1,\ldots ,N}$ is generated by permuting separately {_*X*_1[*n*]_}*n* = 1, …, *N*_ and {_*X*_2[*n*]_}*n* = 1, …, *N*_. Hence, applying LaDynS to ${\left\{X_{1\left[n\right]}^{*},X_{2\left[n\right]}^{*}\right\}}_{n=1,\ldots ,N}$ yields estimates of canonical precision matrix ${\hat{\Omega }}^{*}$, canonical weights ${ŵ}_{k}^{*}\left(t\right)$s, empirical covariance of the estimated latent variables ${\bar{\Sigma }}^{*}=\bar{\mathrm{Var}}\left({ŵ}_{1}^{*\left(1\right)⊤}X_{1}^{*\left(1\right)},\ldots ,{ŵ}_{2}^{*\left(T\right)⊤}X_{2}^{*\left(T\right)}\right)$, and de-sparsified precision matrix estimate ${\tilde{\Omega }}^{*}$ (Equation 13) under the global null hypothesis of no correlated activity. Repeating the bootstrap simulation *B* times produces *B* bootstrap values ${\hat{\Omega }}^{b}$, ${ŵ}_{k}^{b\left(t\right)}$, ${\bar{\Sigma }}^{b}$, and ${\tilde{\Omega }}^{b}$, *b* = 1, …, *B*. We then estimate $\mathrm{Var}\left[{\tilde{\Omega }}_{12}^{\left(t,s\right)}\right]$ with $\hat{\mathrm{Var}}\left[{\tilde{\Omega }}_{12}^{\left(t,s\right)}\right]$, the sample standard error of ${\left\{{\tilde{\Omega }}_{12}^{b,\left(t,s\right)}\right\}}_{b=1,2,\ldots ,B}$. Notice that $\hat{\mathrm{Var}}\left[{\tilde{\Omega }}_{12}^{\left(t,s\right)}\right]$ is obtained under the global null $H_{0}^{\left(t,s\right)}:\Omega_{12}^{\left(t,s\right)}=0$ simultaneously for all (*t, s*), because it is not trivial to simulate bootstrap data that satisfy a specific $H_{0}^{\left(t,s\right)}$ without assuming that all other elements of Ω_12_ are also null. We garnered from simulations that $\hat{\mathrm{Var}}\left[{\tilde{\Omega }}_{12}^{\left(t,s\right)}\right]$ is thus likely to slightly underestimate $\mathrm{Var}\left[{\tilde{\Omega }}_{12}^{\left(t,s\right)}\right]$, which makes for slightly sensitive *p*-values.

*Control of false discoveries:* because we perform tests for many entries of Ω_12_, we cap the false discovery rate


$$\begin{array}{l} \text{FDR}=E\left[\text{FDP}\right],\;\text{where}\;\text{FDP}=\frac{\#\left\{\text{falsely discovered entries}\right\}}{\#\left\{\text{discovered entries}\right\}\vee 1} \end{array}$$ (16)

below a pre-specified level α_BH_ using the procedure of (Benjamini and Hochberg, 1995; BH). To proceed, let $p^{\left[1\right]}\leq \cdots \leq p^{\left[n_{\text{roi}}\right]}$ denote the ordered permutation bootstrap *p*-values *p*^(*t, s*)^ that correspond to *n*_roi_ cross-precision elements in the region of interest. Then, we find the maximum *k*_BH_ satisfying $p^{\left[k_{\text{BH}}\right]}\leq \frac{k_{\text{BH}}}{n_{\text{roi}}}\alpha_{\text{BH}}$ and reject $H_{0}^{\left(t,s\right)}$ with *p*^(*t, s*)^ smaller than $\frac{k_{\text{BH}}}{n_{\text{roi}}}\alpha_{\text{BH}}$. The FDR guarantee is established by Benjamini and Hochberg (1995)) as long as the *p*^(*t, s*)^'s are independent and valid *p*-values.

*Cluster-wise inference by excursion test:* as a further safeguard against falsely detecting correlated activity between brain areas, we obtain *p*-values for each identified connectivity epoch using the excursion test of Ventura et al. (2005)), as follows.

After computing *p*-values *p*^(*t, s*)^ for each precision entry (Equation 15), we define a cluster as any contiguous set of entries whose *p*-values fall below $\frac{k_{\text{BH}}}{n_{\text{roi}}}\alpha_{\text{BH}}$. No additional cluster-forming threshold or minimum cluster size is imposed. For each cluster *k* identified by the BH procedure, we calculate the test statistic:


$$\begin{array}{l} T_{k}:=-2\underset{\left(t,s\right)\in \text{cluster}k}{\sum }\log p^{\left(t,s\right)}, \end{array}$$ (17)

which is reminiscent of Fisher's method Fisher (1925)) for testing the global significance of multiple hypotheses. Large values of *T*_*k*_ provide evidence against cross-area connectivity in cluster *k*, so we calculate the corresponding *p*-value as $\overset{\infty }{\underset{T_{k}}{\int }}f_{T_{\max}}\left(t\right)dt$, where *f*_*T*_max__ is the null distribution of $T_{\max}:=\underset{j}{\max}T_{j}$ under the global null hypothesis of no connectivity anywhere. We use *f*_*T*_max__ rather than the respective null distributions of each *T*_*k*_ to control the family-wise type I error rather than the type I error for each cluster. We approximate *f*_*T*_max__ by the previous permutation bootstrap: for each permuted dataset *b* = 1, …, *B*, we estimate the cross-precision matrix and corresponding *p*-values, identify all clusters of *p*-values below $\frac{k_{\text{BH}}}{n_{\text{roi}}}\alpha_{\text{BH}}$, calculate the corresponding test statistics in Equation 17, and let $T_{\max}^{b}$ be their maximum. The *B* values $T_{\max}^{b}$ are samples from *f*_*T*_max__, which we use to approximate the *p*-value for cluster *k* by the sampling proportion:


$$\begin{array}{l} \frac{1}{B}\overset{B}{\underset{b=1}{\sum }}I\left(T_{\max}^{b}\geq T_{k}\right). \end{array}$$

### 2.5 Locally stationary state-space model and local Granger causality

Our model in Equations 5, 6 can be formulated as a state-space model by rewriting the joint multivariate Gaussian model for the latent vectors in Equation 6 as the set of all conditional distributions


$$\begin{array}{l} \begin{array}{c} Z_{1}^{\left(t\right)}=\overset{d_{\text{auto}}}{\underset{s=1}{\sum }}\alpha_{11,s}^{\left(t\right)}Z_{1}^{\left(t-s\right)}+\overset{d_{\text{cross}}}{\underset{s=1}{\sum }}\alpha_{12,s}^{\left(t\right)}Z_{2}^{\left(t-s\right)}+\eta_{1}^{\left(t\right)},\\ Z_{2}^{\left(t\right)}=\overset{d_{\text{auto}}}{\underset{s=1}{\sum }}\alpha_{22,s}^{\left(t\right)}Z_{2}^{\left(t-s\right)}+\overset{d_{\text{cross}}}{\underset{s=1}{\sum }}\alpha_{21,s}^{\left(t\right)}Z_{1}^{\left(t-s\right)}+\eta_{2}^{\left(t\right)}, \end{array} \end{array}$$ (18)

where *d*_auto_ and *d*_cross_ are the maximal time delays in within-region and cross-region connections, $\eta_{k}^{\left(t\right)}$, *k* = 1, 2, are independent $N\left(0,\phi_{k}^{\left(t\right)}\right)$ random variables, and the $\alpha_{kl,s}^{\left(t\right)}$'s are vector auto-regressive coefficient parameters for the auto-correlation within region if *k* = *l*, *k* = 1, 2, and cross-correlation between regions if *k*≠*l* with time lag *s*.

This state-space formulation is convenient to impose local stationarity on the latent time series, which we do by fitting stationary state-space models in moving windows of time. Local stationarity is justified because the functional connectivity within and between brain regions changes relatively slowly over time. The state-space formulation is also convenient for calculating the Granger causality between regions: *Z*_2_ is said to Granger-cause *Z*_1_ at time *t* if some $\alpha_{12,s}^{\left(t\right)}$ are non-zero (Ombao and Pinto, 2022; and conversely if some $\alpha_{21,s}^{\left(t\right)}$ are non-zero). The coefficient of partial determination (partial *R*^2^) between $\left(Z_{2}^{\left(t-d_{\text{cross}}\right)},\ldots ,Z_{2}^{\left(t-1\right)}\right)$ and $Z_{1}^{\left(t\right)}$, conditional on $Z_{1}^{\left(t-d_{\text{auto}}\right)},\ldots ,Z_{1}^{\left(t-1\right)}$, calculated as


$$\begin{array}{l} R_{2\to 1}^{2}\left(t\right)=1-\frac{\mathrm{Var}\left[\text{residual of Regression 1}\right]}{\mathrm{Var}\left[\text{residual of Regression 2}\right]}, \end{array}$$ (19)

where


$$\begin{array}{l} \begin{array}{l} \text{Regression 1}:Z_{1}^{\left(t\right)}\sim Z_{1}^{\left(t-d_{\text{auto}}\right)}+\cdots +Z_{1}^{\left(t-1\right)}+Z_{2}^{\left(t-d_{\text{cross}}\right)}\\ \;+\cdots +Z_{2}^{\left(t-1\right)},\\ \text{Regression 2}:Z_{1}^{\left(t\right)}\sim Z_{1}^{\left(t-d_{\text{auto}}\right)}+\cdots +Z_{1}^{\left(t-1\right)}, \end{array} \end{array}$$ (20)

may therefore be considered a test statistic for local Granger causality at time *t*. To allow a physiologically meaningful minimum connection time τ_1_ from brain regions 2 to 1, we can also replace the second regression with


$$\begin{array}{l} \begin{array}{l} \text{Regression 2}:Z_{1}^{\left(t\right)}\sim Z_{1}^{\left(t-d_{\text{auto}}\right)}+\cdots +Z_{1}^{\left(t-1\right)}+Z_{2}^{\left(t-\tau_{1}+1\right)}\\ \;+\cdots +Z_{2}^{\left(t-1\right)}\\ \;+\text{1}\left\{\tau_{2}<d_{\text{cross}}\right\}\left(Z_{2}^{\left(t-d_{\text{cross}}\right)}+\cdots +Z_{2}^{\left(t-\tau_{2}-1\right)}\right), \end{array} \end{array}$$

where τ_2_ is the maximum connection time from brain regions 2 to 1, τ_1_ ≤ τ_2_ ≤ *d*_cross_. We set τ_2_ = *d*_cross_ by default, unless there is reason to consider shorter connection times. A plug-in estimator of $R_{2\to 1}^{2}\left(t\right)$ is easily obtained from the estimated covariance matrix of $\left(Z_{1}^{\left(t\right)},\ldots ,Z_{1}^{\left(t-d_{\text{auto}}\right)},Z_{2}^{\left(t-1\right)},\ldots ,Z_{2}^{\left(t-d_{\text{cross}}\right)}\right),$ without actually running the regressions (Anderson-Sprecher, 1994).

Autocorrelations in the latent time series can inflate *R*^2^ values. We therefore test the statistical significance of $R_{2\to 1}^{2}\left(t\right)$ (or $R_{1\to 2}^{2}\left(t\right)$) by comparing its observed value to its null distribution, obtained by repeatedly permuting the trials in one region and calculating $R_{2\to 1}^{2}\left(t\right)$ in the permuted data. We used 2,000 permutations in Section 3.4. The permuted data satisfy the null hypothesis of no cross-region connection and exhibit the same autocorrelation structure as the original latent time series.

## 3 Results

We have introduced LaDynS to estimate the dynamic connectivity between two or more multivariate time series, and we proposed inference procedures to identify when connectivity is statistically significant. We apply LaDynS to experimental data in Section 3.4, but first we present the theoretical results on the equivalence between generative pCCA models and model-free CCA methods. In particular, Theorem 3.3 establishes an equivalence between the GENVAR version of multi-set CCA (Kettenring, 1971) and maximum likelihood applied to our dynamic pCCA. Next we examine its performance on simulated data that have properties similar to the experimental data. The data are simulated from the shared oscillatory driver model described in Section 3.2, which is unrelated to the LaDynS model. Supplementary Section S3.2 contains results based on simulated data that are consistent with the LaDynS model. In Section 3.3, we compare the performance of LaDynS to other existing methods. The reproducible code scripts for the simulations and experimental data analyses are provided at github.com/HeejongBong/ladyns.

### 3.1 Equivalence between dynamic pCCA model and multiset CCA

We first revisit the connection between classical CCA and the probabilistic CCA formulation of Bach and Jordan (2005)). Let ${\left\{\left(X_{1\left[n\right]},X_{2\left[n\right]}\right)\right\}}_{n=1}^{N}$ be independent observations drawn from the joint distribution in Equation 2, and denote the maximum likelihood estimates by $\left({\hat{\beta }}_{1},{\hat{\beta }}_{2}\right)$. The result below restates Theorem 2 of Bach and Jordan (2005)).

**Theorem 0.0.2** (Bach and Jordan, 2005, Theorem 2). *The maximum likelihood estimators (MLEs)* $\left({\hat{\beta }}_{1},{\hat{\beta }}_{2}\right)$ *in* *Equation 2* *based on* *N* *observed vector pairs* {_*X*_1[*n*]_, *X*_2[*n*]_}*n* = 1, 2, …, *N*_ *are equivalent to the CCA solution* $\left({ŵ}_{1},{ŵ}_{2},{\hat{\sigma }}_{cc}\right)$ *in* *Equation 1* *according to:*


$$\begin{array}{l} {\hat{\beta }}_{k}={\bar{\Sigma }}_{kk}{ŵ}_{k}m_{k},\;\text{where }m_{1}m_{2}={\hat{\sigma }}_{cc}\;\text{and }|m_{k}|\leq 1,\;k=1,2. \end{array}$$ (21)

Theorem 3.1 proves that the original CCA setting and the generative pCCA model both yield the same estimate of σ_*cc*_.

We now state an equivalence similar to Theorem 3.1 between the original CCA and the alternative pCCA model.

**Theorem 0.0.3**. *The MLEs* $\left({\hat{\beta }}_{1},{\hat{\beta }}_{2},{\hat{\sigma }}_{12}\right)$ *in* *Equations 3*, *4* *based on* *N* *observed vector pairs* {_*X*_1, [*n*]_, *X*_2, [*n*]_}*n* = 1, 2, …, *N*_ *are equivalent to the CCA solution* $\left({ŵ}_{1},{ŵ}_{2},{\hat{\sigma }}_{cc}\right)$ *according to:*


$$\begin{array}{l} {\hat{\beta }}_{k}={\bar{\Sigma }}_{kk}{ŵ}_{k}m_{k},\;\text{where }m_{1}m_{2}{\hat{\sigma }}_{12}={\hat{\sigma }}_{cc}\;\text{and }|m_{k}|\leq 1,\;k=1,2. \end{array}$$ (22)

In practice we take *m*_1_ = *m*_2_ = 1 out of all possible solutions, because then $Z_{k}|X_{k}={ŵ}_{k}^{⊤}X_{k}$ is the canonical variable almost surely, and σ_12_ = Cov[*Z*_1_, *Z*_2_] equals the canonical correlation σ_*cc*_. This means that σ_12_ is an interpretable parameter, and one for which inference is simpler than for the canonical correlation in the other model, because, in Equation 2, ${\hat{\sigma }}_{cc}$ is an indirect function of the maximum-likelihood parameter estimates (see Theorem 3.1). The interpretability property also persists when we extend Equation 4 to capture the lagged association between two vector time series (see Equation 6). Finally, the choice *m*_1_ = *m*_2_ = 1 implies that the MLEs $\left({\hat{\beta }}_{1},{\hat{\beta }}_{2},{\hat{\sigma }}_{12}\right)$ do not depend on the Gaussian assumption in Equation 3, an assumption that is questionable if, for example, the *X*'s are positive variables like LFP power envelopes or discrete variables like spike counts. Theorem 3.2 is recovered as a special case of Theorem 3.3 below, which extends the result to multiset CCA for vector time series.

We now derive a similar connection between the multiset generalization of CCA introduced by Kettenring (1971)) and the dynamic pCCA model in Equations 5 6. The proof is provided in Supplementary Section S1.

**Theorem 0.0.4**. *Suppose that* ${\hat{\beta }}_{k}^{\left(t\right)},k=1,2,t=1,\ldots ,T$*, and* $\hat{\Sigma }$ *are the MLE in* *Equations 5*, *6* *based on* *N* *observed pairs of vector time series* $\left\{X_{1\left[n\right]}^{\left(t\right)},X_{2\left[n\right]}^{\left(t\right)}:\;t=1,\ldots ,T\right\}$, *n* = 1, …, *N. Then, they are equivalent to the GENVAR multiset CCA solution according to:*


$$\begin{array}{l} {\hat{\beta }}_{k}^{\left(t\right)}=\bar{\mathrm{Var}}\left[X_{k}^{\left(t\right)}\right]{ŵ}_{k}^{\left(t\right)}m_{k}^{\left(t\right)}\text{and }{\hat{\Sigma }}_{kl}^{\left(t,s\right)}= \end{array}$$ (23)

$$\{\begin{array}{ll} 1, & k=l\;and\;t=s,\\ \bar{\text{Var}}[{\hat{Z}}_{k}^{\left(t\right)},{\hat{Z}}_{l}^{\left(s\right)}], & \text{elsewhere,} \end{array}$$

*where the canonical variable is*


$$\begin{array}{l} {Ẑ}_{k}^{\left(t\right)}={ŵ}_{k}^{\left(t\right)⊤}X_{k}^{\left(t\right)}=\frac{1}{m_{k}^{\left(t\right)}}{\hat{\beta }}_{k}^{\left(t\right)⊤}{\bar{\mathrm{Var}}}^{-1}\left[X_{k}^{\left(t\right)}\right]X_{k}^{\left(t\right)}, \end{array}$$ (24)

*and* $|m_{k}^{\left(t\right)}|\leq 1$ *for* *k* = 1, 2 *and* *t*∈[*T*]*. Furthermore, if all* $m_{k}^{\left(t\right)}=1$*, then the MLE minimizes*


$$\begin{array}{l} \log\det\left(\Sigma \right)+\mathrm{tr}\left(\Sigma^{-1}\bar{\Sigma }\right), \end{array}$$ (25)

*where* $\bar{\Sigma }=\bar{\text{Var}}[{\left(\beta_{1}^{(t)⊤}{\bar{\text{Var}}}^{-1}[X_{1}^{\left(t\right)}]X_{1}^{\left(t\right)}\right)}_{t=1,\ldots ,T},$ ${\left(\beta_{2}^{(t)⊤}{\bar{\text{Var}}}^{-1}[X_{2}^{\left(t\right)}]X_{2}^{\left(t\right)}\right)}_{t=1,\ldots ,T}]$*, with* β_1_ *and* β_2_ *scaled such that* $\mathrm{diag}\bar{\Sigma }=\text{1}$.

### 3.2 LaDynS performance on simulated data from a shared oscillatory driver model with time delay

We used a probabilistic model of the inter-areal coherence in local field potentials to generate LFP time series with a dynamic lead-lag relationship between two brain areas. The shared oscillatory driver model with time delay in Ombao and Pinto (2022)) assumes that the coherence between two univariate LFP time series *L*_1_ and *L*_2_ is driven by a univariate latent oscillation *L*_0_ at frequency *f*_0_:


$$\begin{array}{l} \begin{array}{c} L_{k}^{\left(t\right)}=\beta_{k}\cdot L_{0}^{\left(t-\tau_{k}\right)}+\eta_{k}^{\left(t\right)},\;k=1,2, \end{array} \end{array}$$ (26)

where $\eta_{k}^{\left(t\right)}$ is regional baseline noise, and τ_*k*_ is the lead-lag from *L*_0_ to *L*_*k*_. This model generalizes the Synaptic-Source-Mixing (SSM) model of Schneider et al. (2021)), which was shown to be capable of modeling the dynamic cross-regional coherence observed in experimental LFP data between frontal and parietal cortices, as well as between LGN and the visual cortex.

For our simulation, we extended Equation 26 to generate two sets of multi-dimensional LFP time series *L*_*k*_ of dimension *d*_*k*_, *k* = 1, 2, and we used three latent oscillations *L*_0, *j*_, *j* = 1, 2, 3, to allow non-stationary lead-lag relationships between the two brain areas. The resulting model was a special case of the Latent Dynamic Factor Analysis model in Bong et al. (2020)):


$$\begin{array}{l} L_{k}^{\left(t\right)}=\overset{3}{\underset{j=1}{\sum }}\beta_{kj}\cdot L_{0,j}^{\left(t-\tau_{kj}\right)}+\eta_{k}^{\left(t\right)},\;k=1,2, \end{array}$$ (27)

where the factor loadings $\beta_{kj}\in {\mathbb{R}}^{d_{k}}$ were vectors of dimension *d*_*k*_ = 25. We virtually arranged the 25 components of $L_{1}^{\left(t\right)}$ and $L_{2}^{\left(t\right)}$ on 5 × 5 regular two-dimensional arrays so we could create simulated data exhibiting spatial correlations similar to those in the experimental data in Section 3.4. To do that we let $\eta_{k}^{\left(t\right)}$ be spatially correlated Gaussian noise whose temporal power spectrum scales as 1/*f*^α^ for α>1. More specifically, for each temporal frequency *f*, the respective Fourier coefficients ${\hat{\eta }}_{ki}\left(f\right)$ of region *k* and electrode *i* has spatial correlation: $\mathrm{Cov}\left[{\hat{\eta }}_{ki}\left(f\right),{\hat{\eta }}_{kj}\left(f\right)\right]=f^{-\alpha }\exp\left(-\frac{\text{dis}{\text{t}}^{2}\left(i,j\right)}{2\sigma_{\text{spatial}}^{2}}\right)$, where dist(*i, j*) was the distance between electrodes *i* and *j* on one array, α = 1.4, and σ_spatial_ = 0.8.

**Figure 5:**
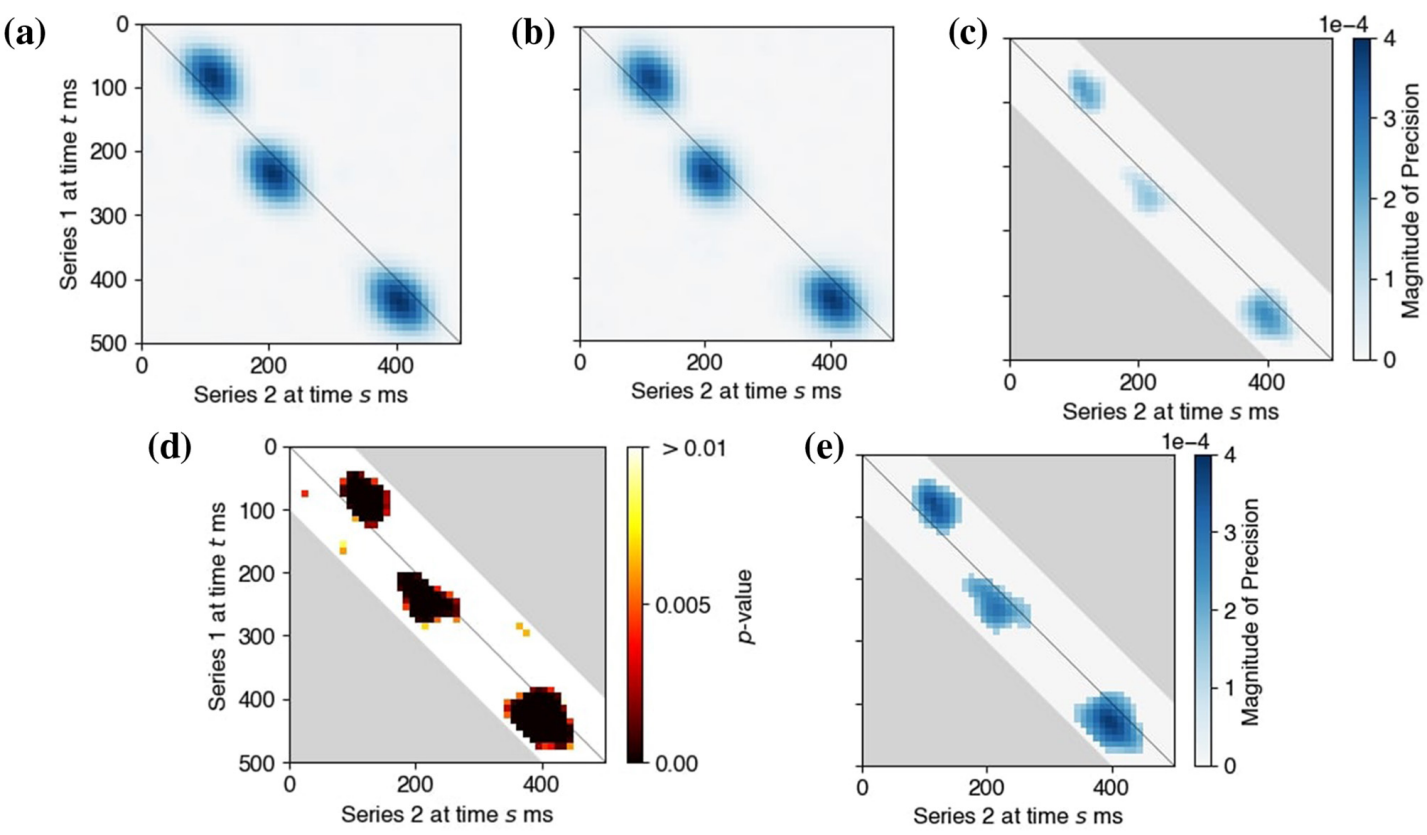
Output and inference of LaDynS applied to simulated datasets from the shared oscillatory driver model. **(a)** True cross-precision matrix Ω_12_ for the connectivity scenario described in Section 3.2 with γ = 0.077. **(b)** Average over 60 simulation datasets of LaDynS de-sparsified precision estimates ${\tilde{\Omega }}_{12}$. There is a good match to the true Ω_12_ in **(a)**. **(c)** Cross-precision estimate ${\hat{\Omega }}_{12}$ for one simulated dataset. It matches **(a)** up to random error. **(d)** Permutation bootstrap p-values for the de-sparsified estimate ${\tilde{\Omega }}_{12}$. **(e)** Discovered non-zero cross-precision estimates by the BH procedure at nominal FDR 5%. The cluster-wise p-values of the three discovered clusters by the excursion test were all smaller than 0.5%. Panels **(a)**, **(b)**, and **(c)** share the same color bar in **(c)**.

Next, we let the factor loadings β_*kj*_ have components $\beta_{kji}=\gamma \cdot \exp\left(-\frac{\text{dis}{\text{t}}^{2}\left(i,p_{kj}\right)}{2\sigma_{\text{spatial}}^{2}}\right)$, *i* = 1, …*d*_*k*_, where *p*_*kj*_ was a randomly chosen location on an array and γ took values in {0.055, 0.063, 0.071, 0.077, 0.084, 0.089}. This set spanned {0.5, 0.66, 0.84, 1.0, 1.16, 1.34} times the observed signal-to-noise ratio (SNR) in the experimental data in Section 3.4. More specifically, the observed data exhibited a signal-to-noise ratio (defined using signal power) of approximately 0.75. Finally, the three latent oscillations *L*_0, *j*_ were set at 18 Hz and designed to induce three epochs of lead-lag relationships between *L*_1_ and *L*_2_ around experimental times 80, 200, and 400 ms; *L*_1_ leads *L*_2_ by 30 ms during the first epoch, and *L*_1_ lagged *L*_2_ by 30 ms during the other two, as depicted in Figure5a.

One simulated dataset consisted of *N* = 1, 000 trials of 500 ms long *d*_1_ = 25 and *d*_2_ = 25-dimensional time series $\left\{L_{1}^{\left(t\right)}\in {\mathbb{R}}^{d_{1}},L_{2}^{\left(t\right)}\in {\mathbb{R}}^{d_{2}}\right\}$ generated from Equation 27 at sampling frequency 1, 000 Hz. We filtered the simulated LFP recordings using the complex Morlet wavelet at frequency *f*_0_ = 18 Hz and bandwidth 50 ms, the same we applied to the data in Section 3.4, and collected the beta oscillation amplitude envelopes as the absolute values of the filtered signals. The complex Morlet wavelet is a complex sinusoidal with a Gaussian envelope (Gabor, 1946), where the bandwidth refers to the Gaussian standard deviation.

(The filtered amplitude at a given time *t* is essentially the amplitude that would be obtained using a harmonic regression with cosine and sine damped by the Gaussian kernel centered at *t*; the wavelet formulation is computationally different and more efficient.)

After downsampling the power envelopes to 100 Hz, we applied LaDynS to the resulting data *X*_1_ and *X*_2_ with *T* = 50 time points.

#### 3.2.1 LaDynS estimation details

The simulated amplitude time series was very smooth, similar to the experimental data. We thus added the regularizer λ_diag_ to the diagonal entries of the estimated correlation matrix $\hat{\Sigma }$ so it could be inverted. Our calibration strategy for λ_diag_ was as follows. Let $X_{k,i}^{\left(t\right)}$ be an observed time series and $S_{k,i}\in {\mathbb{R}}^{T\times T}$ be its auto-correlation matrix, *k*∈{1, 2}, *i*∈{1, …, *d*_*k*_}. Band-pass filtering the LFP data induced auto-correlations in $X_{k,i}^{\left(t\right)}$, which we should observe in $S_{k,i}^{-1}$, unless $S_{k,i}^{-1}$ is degenerate. We thus took λ_diag_ to be such that ${\left(S_{k,i}+\lambda_{\text{diag}}I_{T}\right)}^{-1}$ displayed the expected auto-correlation. Practically, we chose λ_diag_ automatically to minimize the ℓ_2_ distance between the off-diagonal entries of ${\left(S_{k,i}+\lambda_{\text{diag}}I_{T}\right)}^{-1}$ and the band-pass filter kernel-induced auto-correlation, after a scale adjustment, summed over *k* and *i*. Penalizing the diagonal introduced inevitable bias to the sparsified and desparsified LaDynS' precision estimates, $\hat{\Omega }$ and $\tilde{\Omega }$. The other hyperparameters were set to *d*_auto_ = *d*_cross_ = 10, λ_auto_ = 0, and λ_cross_, the penalty on the cross-correlation elements, was determined as per Section 2.3.1.

#### 3.2.2 Results

The simulated data true cross-precision matrix Ω_12_ is unknown so we estimated it by simulation. Given a simulated dataset $\left(X_{1,\left[n\right]}^{\left(t\right)},X_{2,\left[n\right]}^{\left(t\right)}\right)$, for each trial *n* = 1, …, *N* and time *t* = 1, …, *T*, we used Equation 24 to recover the true latent factors $\left(Z_{1,\left[n\right]}^{\left(t\right)},Z_{2,\left[n\right]}^{\left(t\right)}\right)$ from $\left(X_{1,\left[n\right]}^{\left(t\right)},X_{2,\left[n\right]}^{\left(t\right)}\right)$ and known factor loadings $\left(\beta_{1}^{\left(t\right)},\beta_{2}^{\left(t\right)}\right)$. We then obtained the empirical covariance matrix of the true latent factors: $\Sigma^{o}:=\frac{1}{N}\sum Z_{\left[n\right]}Z_{\left[n\right]}^{⊤}$, where $Z_{\left[n\right]}=\left(Z_{1}^{\left(1\right)},\ldots ,Z_{2}^{\left(T\right)}\right)$, and calculated the regularized precision matrix $\Omega^{o}:={\left(\Sigma^{o}+\lambda_{\text{diag}}I\right)}^{-1}$. We estimated the true precision matrix with the average of 200 repeats of Ω^*o*^. Figure 5a shows the cross-regional component $\Omega_{12}^{o}$ of this estimate. We see lead-lag relationships between simulated *X*_1_ and *X*_2_ around 80, 200, and 400 ms, as specified in Section 3.2.

Figure 5c displays the LaDynS cross precision estimate ${\hat{\Omega }}_{12}$ fitted to one dataset simulated under the connectivity scenario depicted in Figure 5a, with connection strength γ = 0.077. Figure 5d shows the permutation bootstrap *p*-values in Equation 15 (with permutation bootstrap simulation size *B* = 200) for the entries of the desparsified cross-precision estimate ${\tilde{\Omega }}_{12}$. Small *p*-values concentrate near the locations of true non-zero cross-precision entries and are otherwise scattered randomly. We then identified the significant connections by first applying the BH procedure with target FDR 5% and next by applying the excursion test at significance level 5% to all discovered clusters (Section 2.4). The significant clusters are plotted in Figure 5e. They match approximately the true clusters in Figure 5a, although they exhibit random variability, as we should expect. To average this random variability out, we estimated ${\tilde{\Omega }}_{12}$ for each of 60 simulated datasets, and plotted their average in Figure 5b. The average LaDynS estimate is a close match to the true cross-precision matrix in Figure 5a.

**Figure 6:**
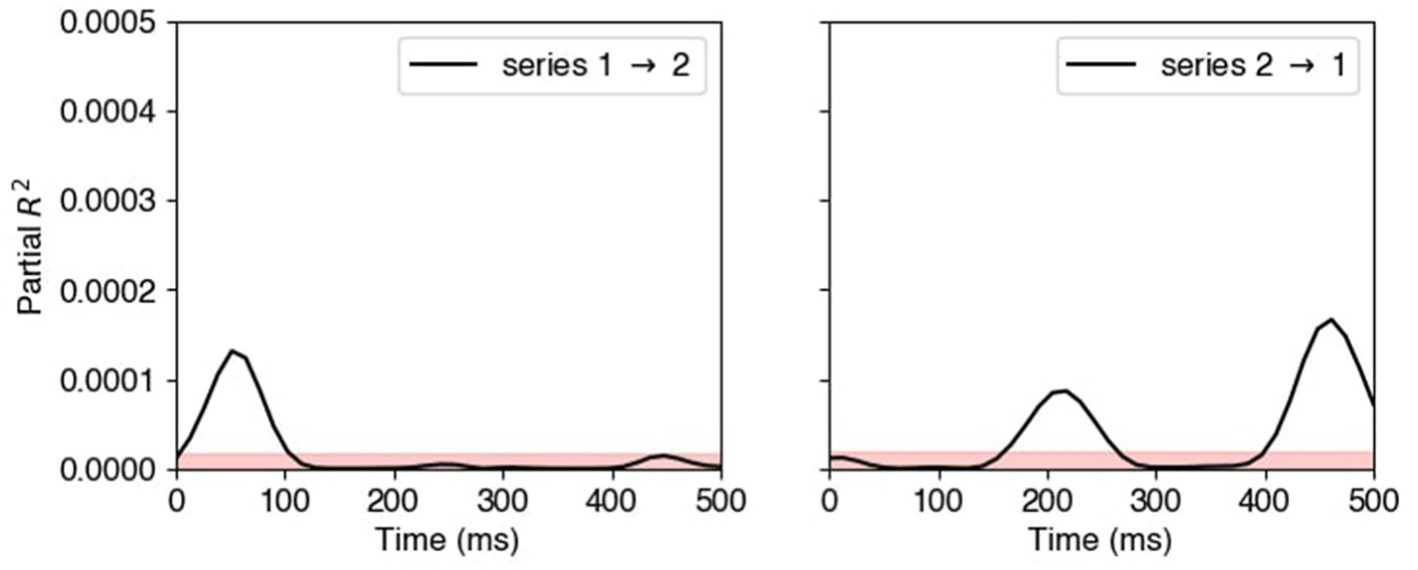
Estimated partial *R*^2^ from locally stationary state-space model

Figure 6 displays the estimated partial *R*^2^ obtained from the locally stationary state-space model described in Section 2.5. The pink shaded areas are pointwise 95th percentiles of the null partial *R*^2^ distribution under the assumption of independence between the two time series. The estimated effects closely reflect the true lead-lag relationships specified in Section 3.2. Specifically, the left panel shows that time series 1 exerts a strong Granger causal influence on time series 2 during the first connectivity epoch around 80 ms, and the right panel shows that time series 2 Granger causes time series 1 during the later connectivity epochs around 200 and 400 ms.

**Figure 7:**
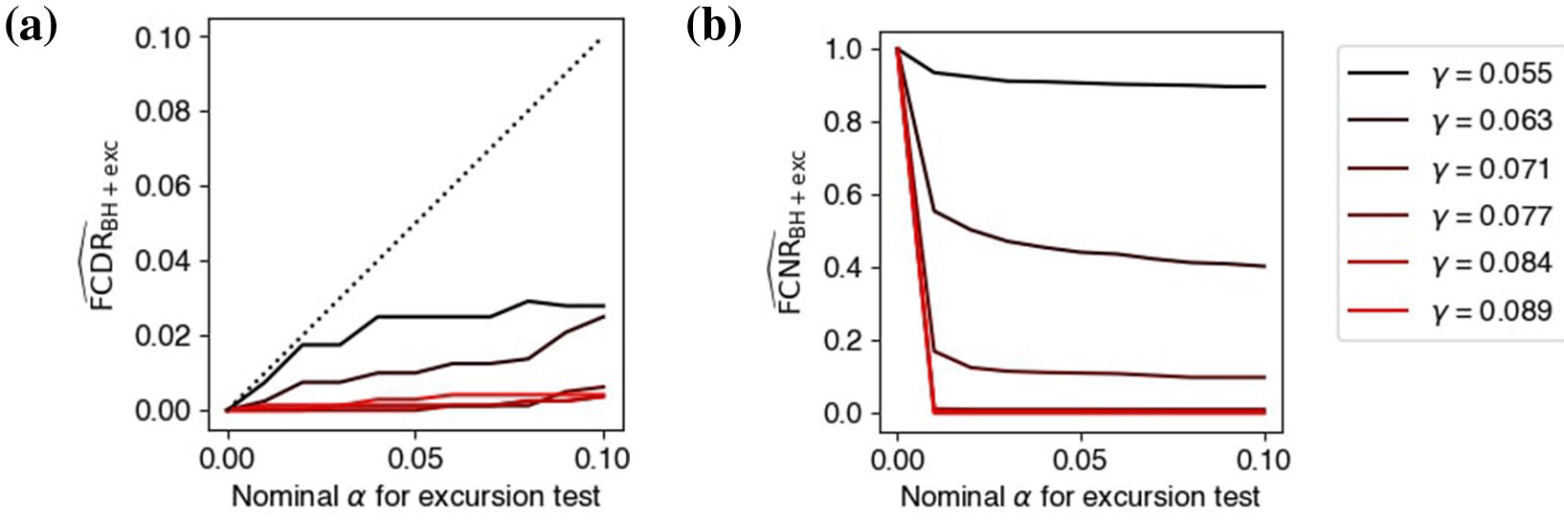
Performance of cluster-wise inference after excursion test. **(a)** False cluster discovery rates and **(b)** false cluster non-discovery rate of BH at target FDR 5% followed by excursion test at significance level α∈[0, 0.10] to identify non-zero partial correlations, under the connectivity scenario in Figure 5a, for the simulated range of connectivity intensities.

Figures 5, 6 used connection strength γ = 0.077. We repeated the simulation for a range of γ such that the SNR in the simulated data beta band ranged from zero to twice the estimated SNR of the experimental data in Section 3.4. Instead of showing graphs, we summarized the performance of LaDynS using false *cluster* discovery and non-discovery rates (Perone Pacifico et al., 2004; FCDR, FCNR) in the estimated precision matrix. We defined a cluster of estimated non-zero precision elements to be falsely discovered if it contained no true effect, and a true cluster was deemed falsely non-discovered if no estimated cluster overlapped with it. We estimated these rates with the corresponding proportions in 60 simulated datasets. They are plotted in Figure 7 against nominal significance levels of the excursion test between 0 and 10%, where the target FDR for the BH procedure was fixed at 5%. The FCDR remained below the nominal level across all tested significance values and connectivity intensities (all or almost all discovered clusters were true clusters) and for large enough γ, the FCNR was zero (all clusters were discovered).

Perhaps reporting FDR and FNR would have been more informative, but we reported FCDR and FCNR because they were less sensitive to Ω_12_ being approximated rather than known in this particular example. See Supplementary Section S3.2 for an example where Ω_12_ is known, so FDR and FNR could be calculated.

*Remark*. A defining feature of LaDynS is that it is capable of estimating amplitude lead-lag relationships between two high-dimensional oscillatory time-series even if there is no other source of correlations, such as coherence or phase locking, between them. We demonstrated this in Supplementary Section S3.1 using simulated data from the model in this section, modified so it would induce amplitude lead-lag relationships without inducing phase correlations between the simulated pair of time-series.

**Figure 8:**
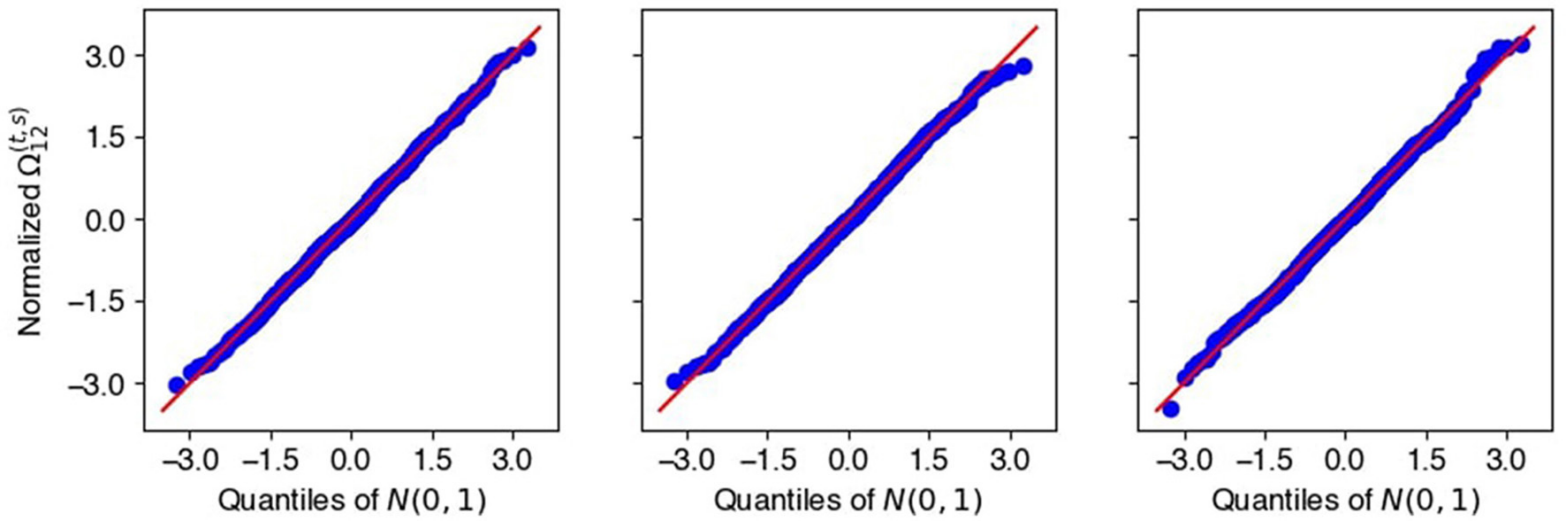
Null distributions of three representative entries of ${\tilde{\Omega }}_{12}^{\left(t,s\right)}/\sqrt{\hat{\mathrm{Var}}\left[{\tilde{\Omega }}_{12}^{\left(t,s\right)}\right]}$, obtained from *R* = 60 simulated datasets (Section 3.2) and compared to the standard Gaussian distribution via QQ-plots. The good agreement suggests that the Normal assumption in Equation 13 is appropriate.

*Checking the assumptions in* *Equations 14*, *15*. We assumed that, under the null hypothesis $H_{0}^{\left(t,s\right)}:\Omega_{12}^{\left(t,s\right)}=0$, the desparsified precision entries ${\tilde{\Omega }}_{12}^{\left(t,s\right)}$ were normally distributed and $\mathrm{Var}\left[{\tilde{\Omega }}_{12}^{\left(t,s\right)}\right]$ was well approximated by the permutation bootstrap variance $\hat{\mathrm{Var}}\left[{\tilde{\Omega }}_{12}^{\left(t,s\right)}\right]$ . To check the former, we compared the empirical distribution of *R* = 60 repeat estimates ${\tilde{\Omega }}_{12}^{\left(t,s\right)}/\sqrt{\hat{\mathrm{Var}}\left[{\tilde{\Omega }}_{12}^{\left(t,s\right)}\right]}$ to the standard normal distribution via QQ-plots. Figure 8 shows QQ-plots for three randomly chosen time pairs (*t, s*) that satisfy $\Omega_{12}^{\left(t,s\right)}=0$. The good agreement between empirical and theoretical quantiles suggests that the normal assumption is appropriate.

Next, for each (*t, s*) such that $H_{0}^{\left(t,s\right)}:\Omega_{12}^{\left(t,s\right)}=0$, we checked the validity of the permutation bootstrap variance estimate $\hat{\mathrm{Var}}\left[{\tilde{\Omega }}_{12}^{\left(t,s\right)}\right]$ by comparing it to the empirical variance of the *R* = 60 estimates ${\tilde{\Omega }}_{12}^{\left(t,s\right)}$.

**Figure 9:**
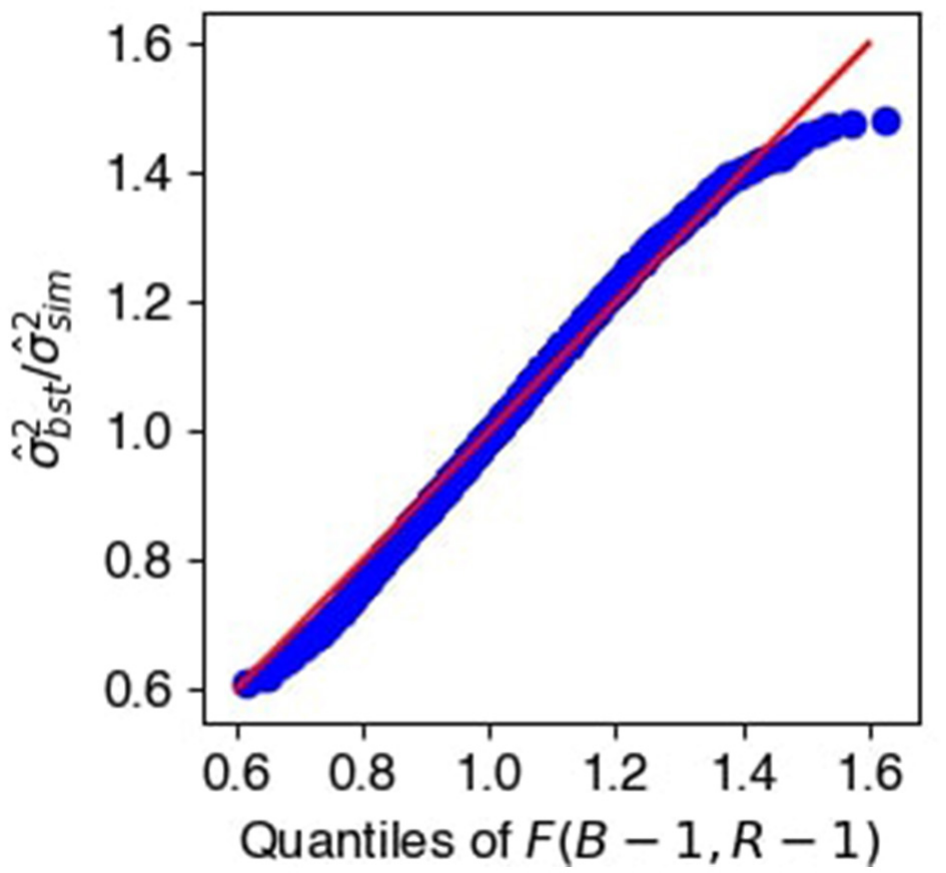
Standard deviations of desparsified precision elements. *F*-statistics of ratios between bootstrap and empirical variances for the null entries of Ω_12_, showing good agreement.

Figure 9 shows the Q-Q plot of their ratios against the quantiles of the *F*(*B*−1, *R*−1) distribution, which suggests that the two estimates are equal up to random error.

### 3.3 Comparison to existing methods

**Figure 10:**
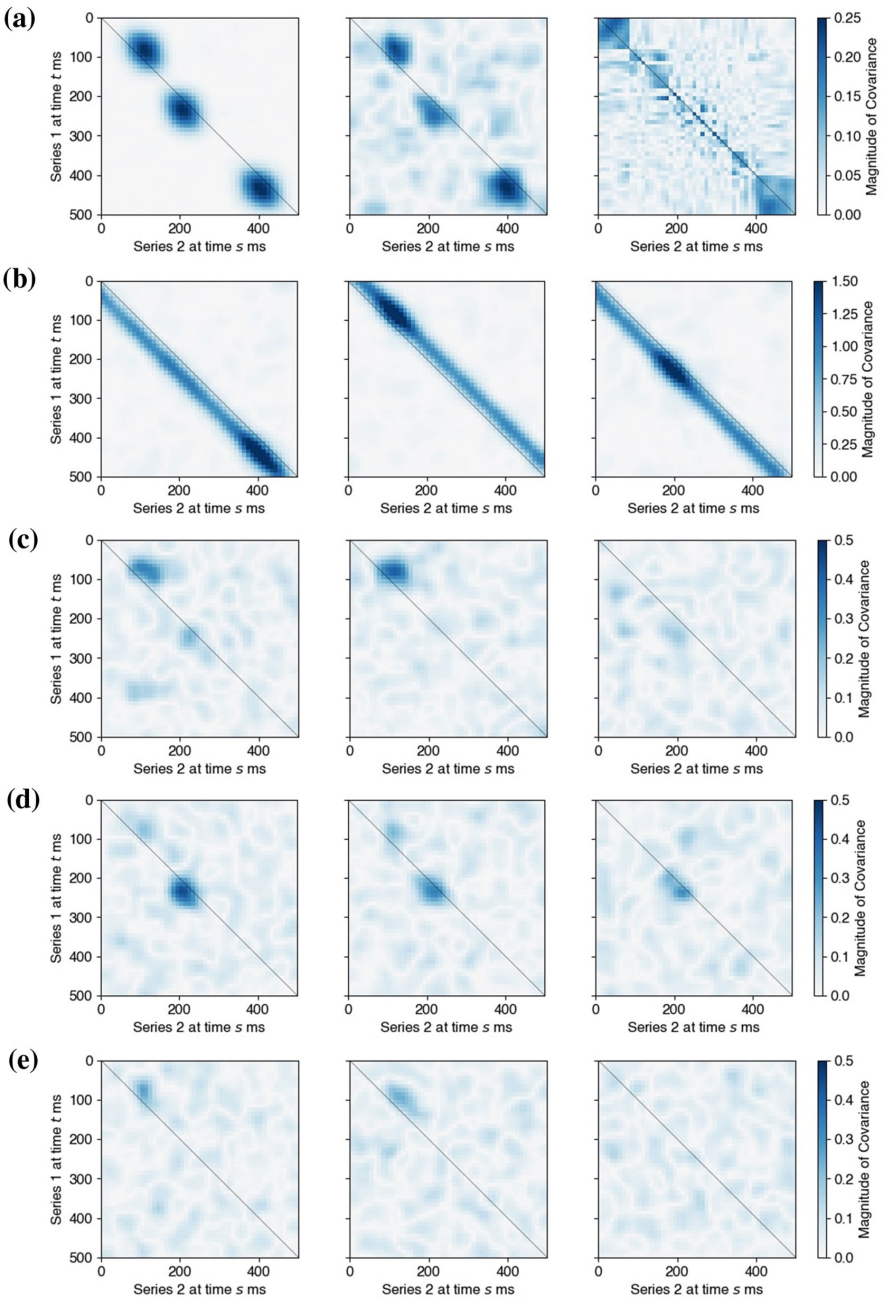
Comparison of LaDynS and competing methods applied to one simulated dataset from the shared oscillatory driver model under the three-factor scenario. **(a)** (**left**) The true cross-covariance matrix. (**center and right**) Estimated cross-covariance matrices from LaDynS and DKCCA, respectively. **(b)** The cross-covariance estimates obtained by DLAG, which pairs the three factors in region 1 with the three factors in region 2, yielding three cross-covariance plots (one for each factor pair). **(c–e)** The cross-covariance estimates obtained by GPFA, which considers all possible between-region factor pairs among the three factors in region 1 and the three factors in region 2, yielding nine cross-covariance plots.

**Figure 11:**
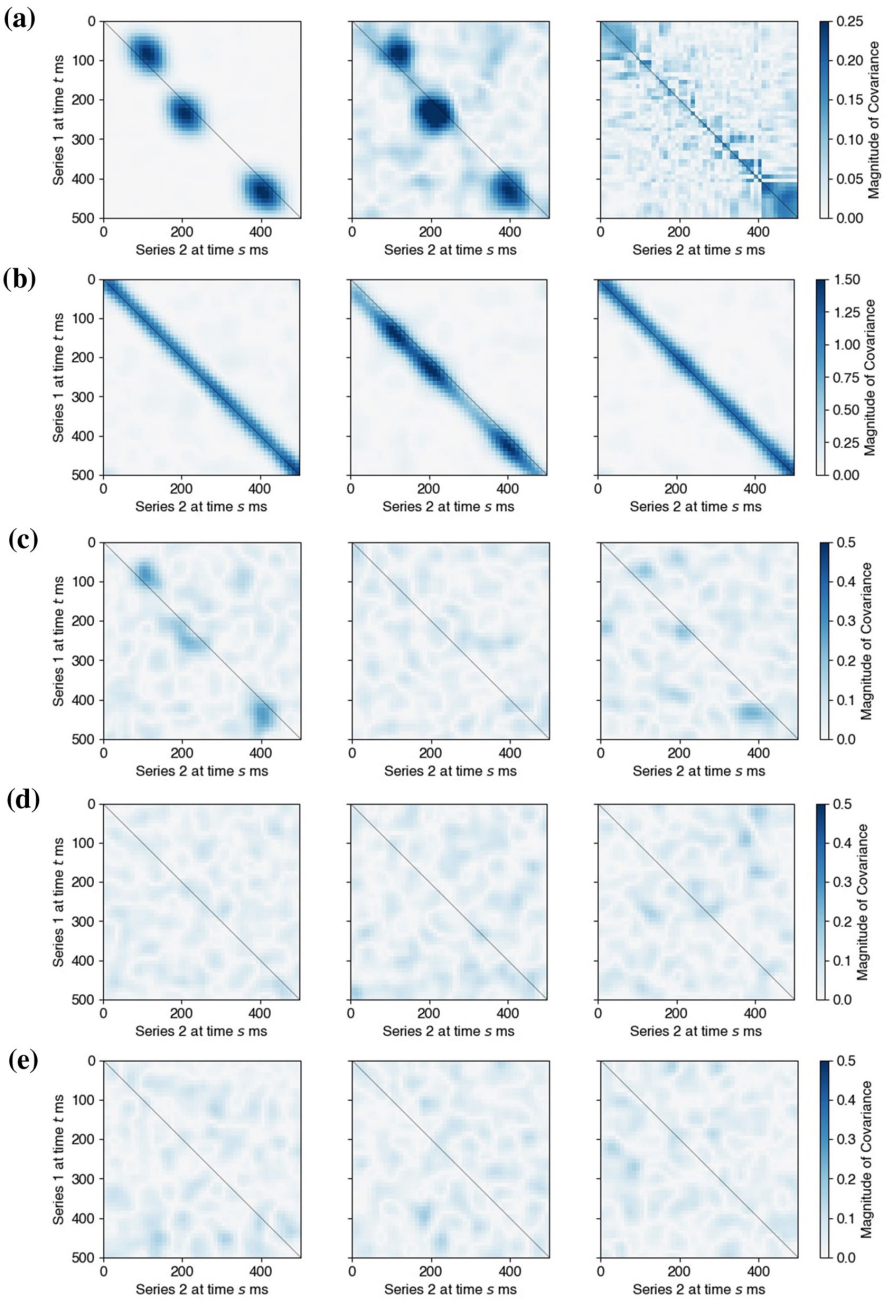
Comparison of LaDynS and competing methods applied to one simulated dataset from the shared oscillatory driver model under the single-factor scenario. **(a)** (**left**) The true cross-covariance matrix. (**center and right**) Estimated cross-covariance matrices from LaDynS and DKCCA, respectively. **(b)** The cross-covariance estimates obtained by DLAG, which pairs the three factors in region 1 with the three factors in region 2, yielding three cross-covariance plots (one for each factor pair). **(c–e)** The cross-covariance estimates obtained by GPFA, which considers all possible between-region factor pairs among the three factors in region 1 and the three factors in region 2, yielding nine cross-covariance plots.

In this section, we compare the performance of LaDynS on simulated data generated from the shared oscillatory driver model (see Section 3.2) with three existing methods: Dynamic Kernel Canonical Correlation Analysis (DKCCA; Rodu et al., 2018), Delayed Latents Across Groups (DLAG; Gokcen et al., 2022), and Gaussian Process Factor Analysis (GPFA; Yu et al., 2009). The simulated data are generated under two distinct connectivity scenarios:

- *Three-factor scenario*: three pairs of latent factors drove the connections, each active in a distinct time epoch, as depicted by the true cross-covariance matrix in Figure 10a, left panel. Each pair had a unidirectional influence with a fixed lead-lag.
- *Single-factor scenario*: the epochs and lead-lags of the connections were the same as in the three-factor scenario (Figure 11a, left panel), but this time a single pair of latent factors drove all three epochs of connections. As a result, the connection between the pair of latent factors was bidirectional, and the lead-lag changed over time.

LaDynS models each latent factor time series $Z_{k}^{\left(t\right)}$ to have time-varying factor loadings $\beta_{k}^{\left(t\right)}$, accommodating changes in the activated latent factors across time *t*. Similarly, DKCCA allows each canonical component time series to have time-varying canonical weights. As a result, both LaDynS and DKCCA can capture multiple factor-driven connections using only a single pair of latent factors or canonical components, provided these underlying factors are active in distinct time intervals (e.g., the three-factor scenario). By contrast, DLAG and GPFA use fixed factor loadings over time, and thus require three latent factors per set of observed time series to capture the same three-factor scenario. Accordingly, we estimated the interaction between two sets of simulated time series, $X_{1}^{\left(t\right)}$ and $X_{2}^{\left(t\right)}$, under the following configurations:

- LaDynS: we followed the estimation details in Section 3.2.1. Although LaDynS represents the between-sets interaction via the cross-precision matrix Ω_12_, we use the cross-covariance matrix Σ_12_ here to facilitate comparison with the other methods, which estimate cross-covariance matrices only.
- DKCCA: we estimated the cross-covariance between a single pair of canonical components defined by a sliding time window of width 9 (*g* = 4 in the original notation).
- DLAG: we used three between-group and two within-group factors for each set of time series. In DLAG, each between-group factor in one set is exclusively paired with a counterpart in the other set, yielding three cross-covariance matrix estimates.
- GPFA: because GPFA inherently models a single set of time series, it does not directly provide a cross-connection component. We therefore used a two-step approach: first, we fitted GPFA with three latent factors to each of $X_{1}^{\left(t\right)}$ and $X_{2}^{\left(t\right)}$; then, we computed cross-covariance matrices for all 3 × 3 pairs of latent factors, producing nine cross-covariance estimates.

For all other tuning hyperparameters of DKCCA, DLAG, and GPFA, we used the default settings provided in the example scripts of the respective code packages. For details, we refer the reader to the example code in our ladyns package.

Figure 10 presents the simulation results for the three-factor scenario. Both LaDynS and DLAG successfully identified all three interaction epochs (Figure 10a, center panel, and Figure 10b), whereas GPFA only captured the first two (Figure 10c–e), and DKCCA failed to detect the interaction entirely (Figure 10a, right panel). This outcome for GPFA is unsurprising, given that GPFA was designed primarily to capture dominant within-set dynamics rather than cross-set interactions. Consequently, if cross-regional interactions are relatively weak compared to the within-region variability (as in this three-factor scenario), GPFA may fail to detect all active connections.

Under the single-factor scenario, where the interaction between a single pair of latent factors is sufficiently strong, GPFA was able to recover all three interaction epochs (Figure 11c–e), matching the performance of LaDynS (Figure 11a, center panel). By contrast, DLAG struggled to capture the bidirectional nature of the interaction (Figure 11b), consistent with its assumption that each pair of between-group latent factors has a fixed lead-lag relationship. We additionally ran DLAG with a single between-group latent factor to match the true latent dimensionality in this scenario; however, its qualitative behavior remained unchanged, and DLAG continued to struggle to capture the bidirectional nature of the interaction. DKCCA again did not detect any meaningful interaction in this setting.

Overall, LaDynS was the only method that consistently identified all three interaction epochs in both connectivity scenarios. DKCCA showed weak performance in both scenarios, while DLAG and GPFA had diminished power in at least one of the scenarios. The performances of DLAG and GPFA would further degrade if we did not specify the correct number of latent variables.

### 3.4 Experimental data analysis

We applied LaDynS to local field potentials (LFPs), collected in the experiment described in Khanna et al. (2020)), from two Utah arrays implanted in a Macaque monkey's prefrontal cortex (PFC) and V4 during a memory-guided saccade task. Each trial of the task started with a monkey fixating its eyes on a dot at the center of the screen. A visual cue was given for 50 ms to indicate a target location, which was randomly chosen from 40 candidate locations (eight directions and five amplitudes) that tiled the display screen. The cue was turned off and the monkey had to remember the target location while maintaining eye fixation for a delay period of 500 ms. After the delay period, the monkey made a saccade to the remembered position, and a liquid reward was provided on successful trials. As in Khanna et al. (2020)), we analyzed the time series during the delay period, based on 3, 000 successful trials. Time *t* = 0 corresponds to the start of that period. The data are available in Snyder et al. (2022)).

Because beta oscillations are often associated with communication across brain areas (Klein et al., 2020; Miller et al., 2018), we filtered LFP recordings at a beta oscillation frequency 18 Hz and obtained the beta oscillation power envelopes as described in Section 3.2.1. We chose 18 Hz because it was the frequency having the largest power within the range 12–40 Hz (see Supplementary Figure S11). After downsampling the power envelopes to 200 Hz, we applied LaDynS with the regularizer λ_diag_ on the diagonal of Σ, as in Section 3.2.1, because the filtered data were very smooth.

**Figure 12:**
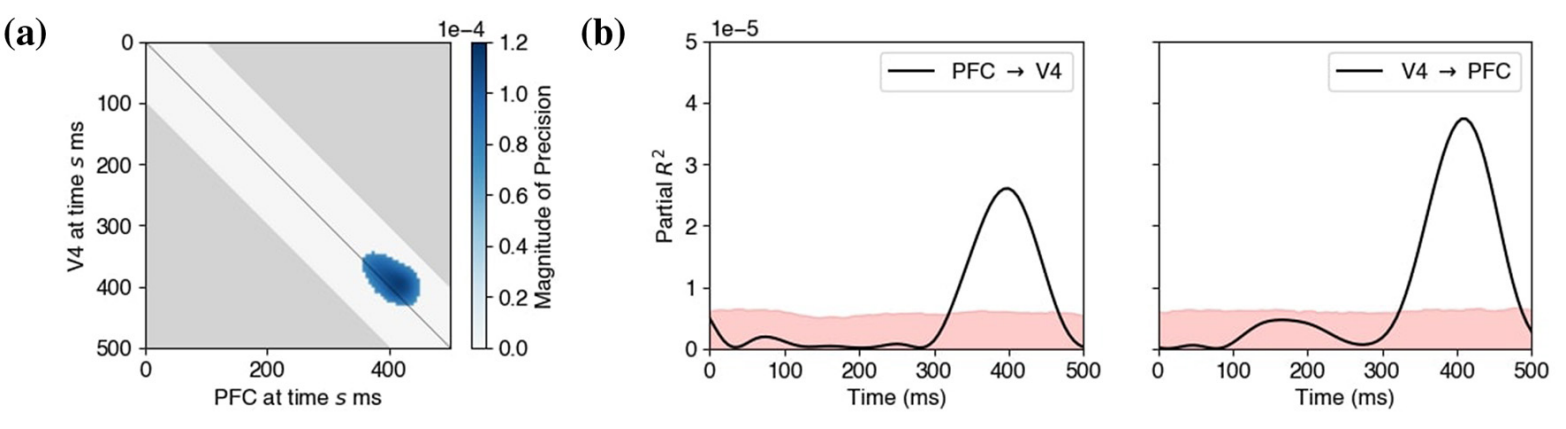
Experimental data analysis. **(a)** LaDynS inference. Discovered region of cross-precision using BH at nominal FDR 5% and the excursion test (*p* < 0.0005). The light gray area shows the region of time considered (one area leading the other by at most 100 ms). The blue blob suggests that activity in V4 preceded that in PFC around 400 ms in the delay period. **(b)** Estimated partial *R*^2^ from locally stationary state-space model for (**left**) PFC → V4 and (**right**) V4 → PFC. The pink shaded areas indicate the range of values that fall below the 95th percentile under the null hypothesis of independence between V4 and PFC.

Figure 12a shows the only epoch of significant contiguous region of the precision matrix identified by our method (FDR at 5%, *p* < 0.0005 by the excursion test in Section 2.4). This result provides strong evidence that the 18 Hz beta amplitudes in PFC and V4 were correlated (after conditioning on beta amplitudes at all other times and lags) around 400 ms after the start of the delay period.

**Figure 13:**
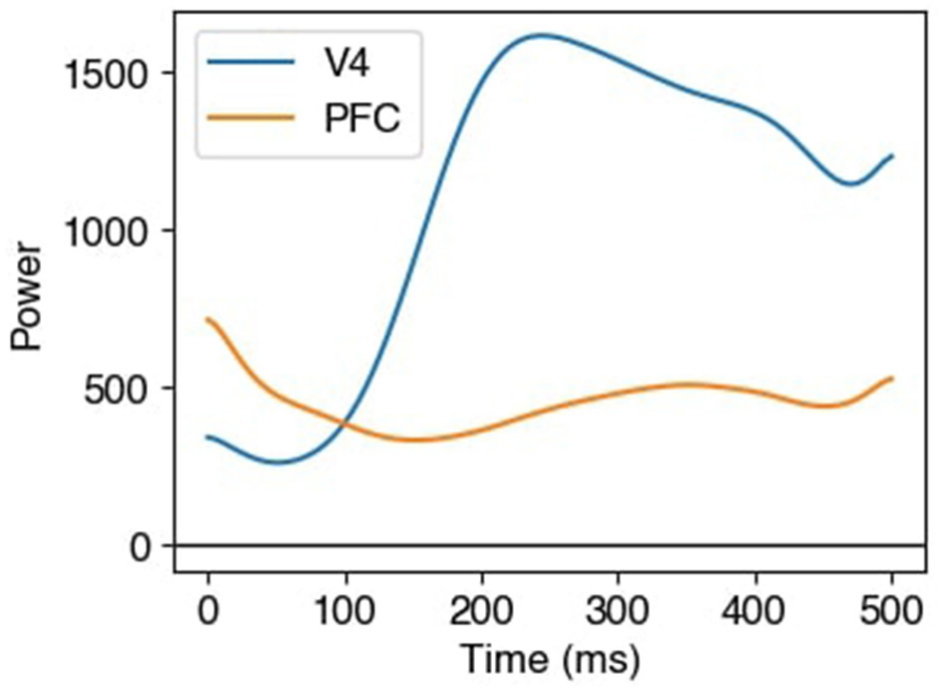
Estimated beta powers of electrophysiological activity driven by latent factors in V4 and PFC as functions of time. The summed beta power in the data $X_{k}^{\left(t\right)}$ attributable to the latent time series at time *t* was estimated by the ℓ_2_ norm of the factor loading vector $\beta_{k}^{\left(t\right)}$; see Equation 5.

To better understand this relationship, we used the estimated latent time series to compute partial *R*^2^ values under an assumption of locally stationarity, applying the model in Equation 18, Section 2.5. The frequency 18 Hz corresponds to a period of 55 ms, and it is hard to detect non-stationarity at time scales finer than a few periods. We therefore used a moving window of 100 ms (i.e., larger than the period) to calculate partial *R*^2^, allowing connection delays between V4 and PFC in the τ_1_ = 15 to τ_2_ = 30 ms range. The partial *R*^2^ from PFC to V4 and from V4 to PFC are shown in Figure 12b. There are large excursions of *R*^2^ above the null values in both plots, suggesting that, at around 400 ms post stimulus, the two areas are involved in a bidirectional network: the power of the activity in each area predicts the power of the oscillation in the other, following a short delay (after conditioning on the power at all other times and lags). To see that this is not due solely to the activity passing through the visual stream, in Figure 13, for each of the two latent time series, we plotted the estimated total beta power as a function of time. The power in V4 increases dramatically much earlier than 400 ms, starting around 100 ms and reaching a peak just after 200 ms; it then remains substantial during the remainder of the delay period. The results in Figure 12 can not be explained by those in Figure 13 alone.

**Figure 14:**
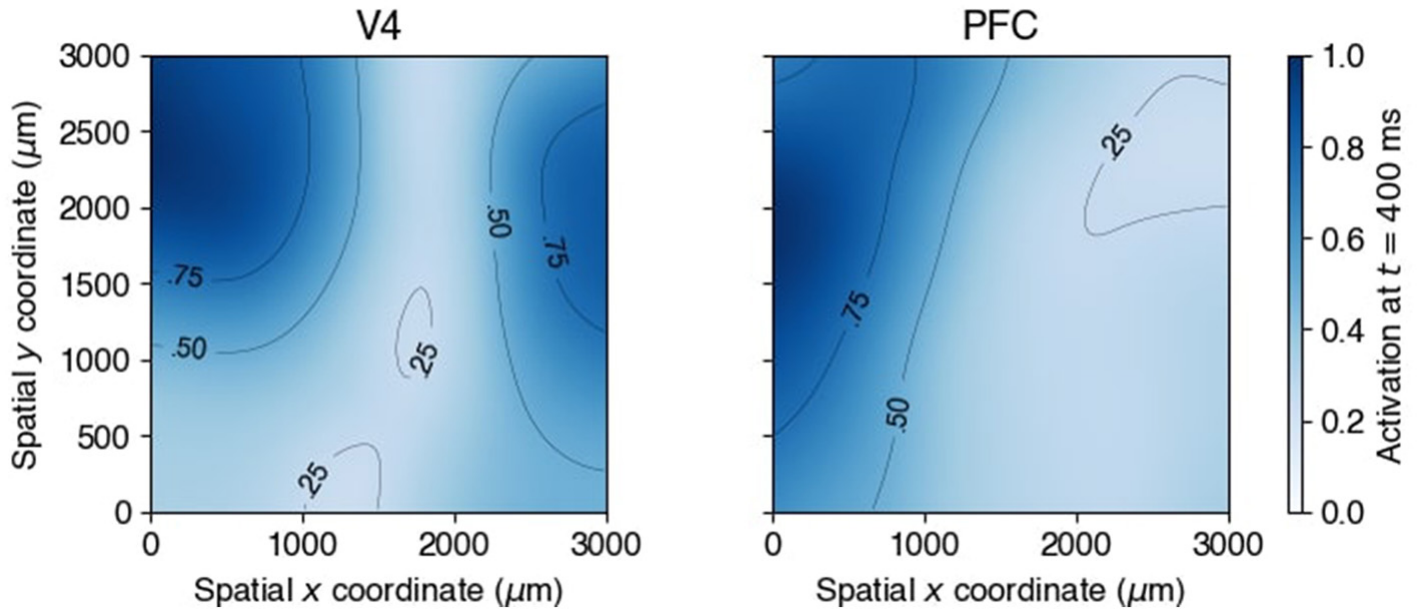
Factor loadings of V4 and PFC, spatially smoothed, normalized, and color coded over the electrode coordinates (μ*m*) on **(left)** V4 and **(right)** PFC at late delay period (400 ms). Contours at 0.25, 0.5 and 0.75 of the maximal power were added.

It is also possible to get spatial information from the normalized factor loadings across the electrode arrays, which are displayed in Figure 14 for experimental time 400 ms. The loadings were rescaled by their maximal value across the array. (An animation over the complete timeline is available at github.com/HeejongBong/ladyns.) It is apparent that a relatively small proportion of the electrodes, residing in limited portions of the recording areas, contribute most of the beta oscillatory power identified by the bivariate time series.

## 4 Discussion

To describe cross-population interactions of oscillatory amplitudes in high-dimensional neural field potentials, we developed a novel and intuitive procedure: in LaDynS, cross-population interactions are described using the cross-correlation of latent drivers. To arrive at a statistically rigorous approach, we provided a time-series extension of probabilistic CCA together with a novel sparse estimation methodology. According to our Equation 5, each of the two multivariate time series is driven by a single latent time series, with the cross-dependence of these two latent time series representing cross-region interaction. According to Equation 6, the latent bivariate time series is a discrete Gaussian process, but its correlation matrix is unrestricted, allowing for time-varying dynamics, i.e., statistical non-stationarity. The repeated trial structure enabled us to estimate the resulting high-dimensional covariance matrix by applying sparse estimation and inference methods. We found, and displayed in Figure 12b, an interesting interaction between PFC and V4, involving beta power, that appeared during the late delay period, with the interaction being bidirectional. The results were based on partial *R*^2^ values, computed from the estimated covariance matrices, corresponding to lagged regressions of one latent time series on the other. The analysis in Figure 12b is in the spirit of Granger causality, but differs from it by allowing for non-stationarity, so that we could obtain the time-varying results.

The partial *R*^2^ values in Figure 12b are highly statistically significant, but they are very small. We note that our diagonal regularization of the estimated latent precision matrix artificially reduced these values, so that the scale is no longer interpretable in familiar terms. The cross-correlation estimates are several orders of magnitude larger (see Supplementary Figure S12). However, they remain smaller than .1, as are the raw correlations at 400 and 425 ms, respectively, across individual electrodes in V4 and PFC (not shown). This reflects the highly inhomogeneous nature of the LFP voltage recordings together with the dominance of low frequencies (unfiltered LFP spectra typically follow 1/*f*^α^ trends, where *f* is frequency and α is roughly 1.5 to 2; see Kass et al. (2023))). The large number of repeated trials allowed us to find and document the results.

In addition to making the analysis possible, the repeated trial structure suggests substantive interpretation based on trial-to-trial variability. Although investigators take pains to make the experimental setting nearly the same on each trial, the inevitable small fluctuations in the way the subject interacts with the environment, together with changes in the subject's underlying state (involving alterations in motivational drive, for example), lead to observable fluctuations in behavior and in the recorded neural activity. Although the network sources of trial-to-trial variability in the PFC and V4 data are unknown, they produce the kind of correlated activity revealed in Figure 12b. To interpret it, we acknowledge there could be some potentially confounding trial-to-trial variation — such as changing motivational state, stimulus timing, bandpass-filter distortions, and volume conduction — that drive beta power in V4 and PFC, having just the right differential time lags to produce the correlated activity picked up by the partial *R*^2^ values, as cautioned by Lintas et al. (2021)). Could such task-irrelevant pulses of activity change across time, within repetitions of the task, in such a way as to produce the peaks in Figure 12b? It is possible, but it would be surprising, especially when we consider contemporary ideas about beta oscillations during working memory tasks (Miller et al., 2018) along with the well-identified distinction between early and late visual processing that, presumably, corresponds to a distinction between feedforward and recurrent (bidirectional) flow of information (Supèr et al., 2001; Buschman and Miller, 2007; Del Cul et al., 2007; Yang et al., 2019; Chen et al., 2022; Mejias et al., 2016). The alternative we mentioned, that PFC and V4 are involved, together, in goal-directed visual processing and memory, with the two areas acting bidirectionally around 400 ms post-stimulus, seems likely.

There are many ways to extend the ideas developed here. While we applied LaDynS to LFP recordings, the framework is applicable more broadly to other slowly varying multidimensional time series. In particular, the proposed methodology can be extended to other neural modalities such as EEG, MEG, and fMRI. Although data from these modalities often violate global stationarity assumptions underlying classical approaches such as cross-correlograms (Guan et al., 2020; Klonowski, 2009; Chang and Glover, 2010), local stationarity remains reasonable when functional connectivity depends on cognitive/behavioral states that evolve on time scales longer than the sampling interval. While we have not yet explored such extensions empirically, LaDynS is particularly appealing in this regime, as it explicitly accommodates slowly varying connectivity and cross-correlation over time. At the same time, signals in EEG and MEG often reflect mixtures of activity from multiple brain structures and switches between metastable neural states, which may introduce additional confounding and complicate the interpretation of inferred associations.

LaDynS may perform less favorably in settings where within-region noise autocorrelations dominate the signal. The simulation results in Figure 4 of Bong et al. (2020)) illustrate the resulting loss of specificity under such adversarial conditions. In these cases, methods that explicitly model within-region autocorrelated noise, such as DLAG (Gokcen et al., 2022), can outperform LaDynS. In Bong et al. (2020)), we proposed an extension of LaDynS that addresses this issue by allowing the within-region noise processes ϵ_*k*_ to follow general time series structures and by modeling the latent processes driving each brain region as multidimensional. That brief report, which focused on data filtered at a different frequency band, did not provide the methodological details or inferential procedures developed here. An important direction for future study is to generalize the present framework to the setting considered in Bong et al. (2020)).

Another important direction for future research is a theoretical analysis of how regularization hyperparameters affect recovery of the underlying lag structure. In this study, we focused on proposing a regularization-based approach to functional connectivity estimation together with a data-driven hyperparameter calibration scheme and demonstrated its performance through simulation studies, while establishing theoretical guarantees for hyperparameter selection. Optimality remains an interesting topic for future investigation. In addition, developing a theory that accounts for dependence across experimental trials is of particular interest, as such dependence may arise from long-term memory or adaptation effects in neural recordings and could impact the validity of the proposed inference procedure.

Furthermore, for band-pass filtered data, such as those analyzed in Section 3.4, phase analysis (Klein et al., 2020) could be combined with amplitude analysis based on the complex normal distribution, as in Urban et al. (2023)) (which developed a latent variable model at a single time point). Multiple frequencies and non-linear association, such as cross-bicoherence, could be considered, along the lines of Tort et al. (2010)); Gallagher et al. (2017)); Aliramezani et al. (2024)); Abe et al. (2024)), as well. A different direction for additional research would be to simplify the version of LaDynS we have used here by imposing a suitable spatiotemporal structure on the latent time series. Regardless of whether such approaches are fruitful, the general framework of LaDynS could be of use whenever interest focuses on time-varying interactions among groups of repeatedly-observed multivariate neural time series.

## Data Availability

The original contributions presented in the study are included in the article/Supplementary material, further inquiries can be directed to the corresponding author.
