# Supplementary figures and images for: Cross-population amplitude coupling in high-dimensional oscillatory neural time series
Source: Front Comput Neurosci. 2026 Feb 3;20:1703722. doi: 10.3389/fncom.2026.1703722 (PMC12909514; doi:10.3389/fncom.2026.1703722)

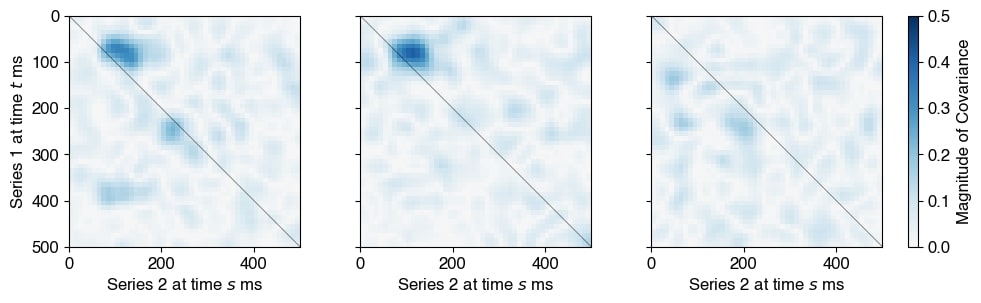

Supplement: Supplementary file 1 [file Data_Sheet_1.ZIP › images/Sighat_10_gpfa_1.jpg]

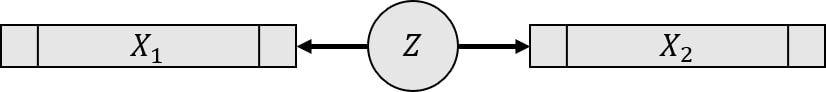

Supplement: Supplementary file 1 [file Data_Sheet_1.ZIP › images/pCCA_Bach_Jordan.jpg]

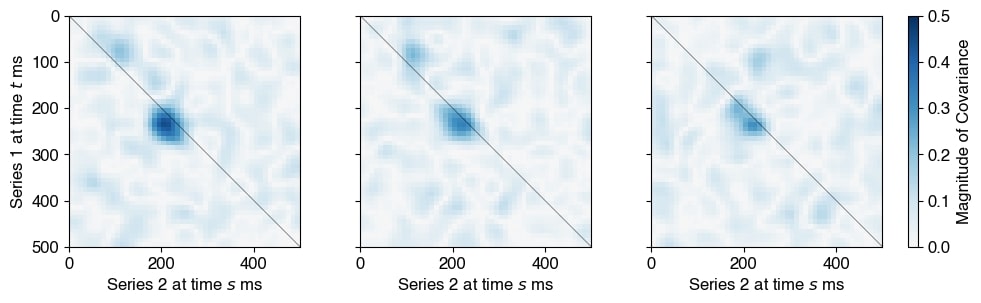

Supplement: Supplementary file 1 [file Data_Sheet_1.ZIP › images/Sighat_10_gpfa_2.jpg]

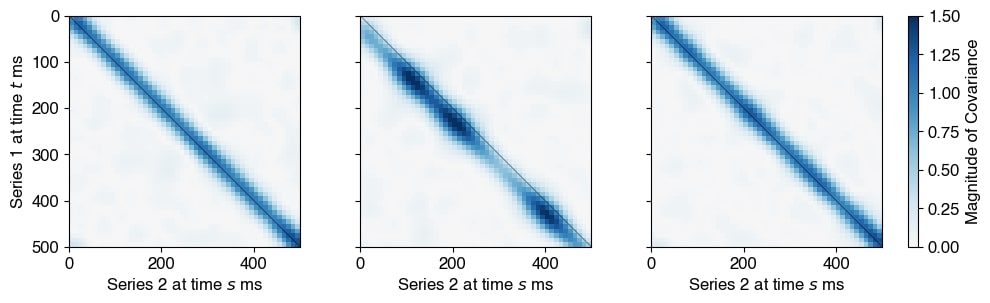

Supplement: Supplementary file 1 [file Data_Sheet_1.ZIP › images/Sighat_11_dlag.jpg]

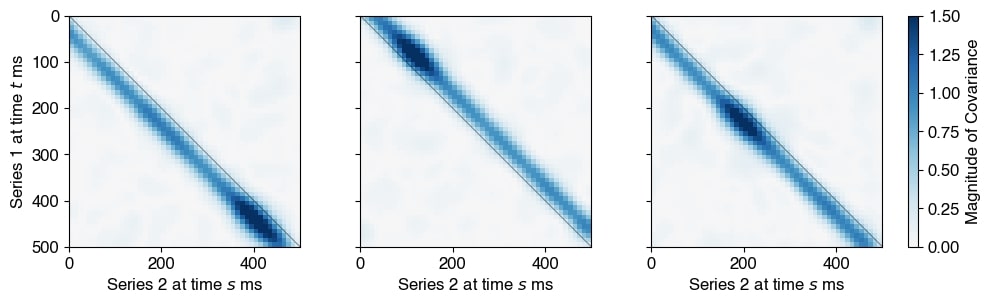

Supplement: Supplementary file 1 [file Data_Sheet_1.ZIP › images/Sighat_10_dlag.jpg]

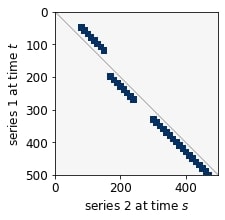

Supplement: Supplementary file 1 [file Data_Sheet_1.ZIP › images/groundtruth_SSM.jpg]

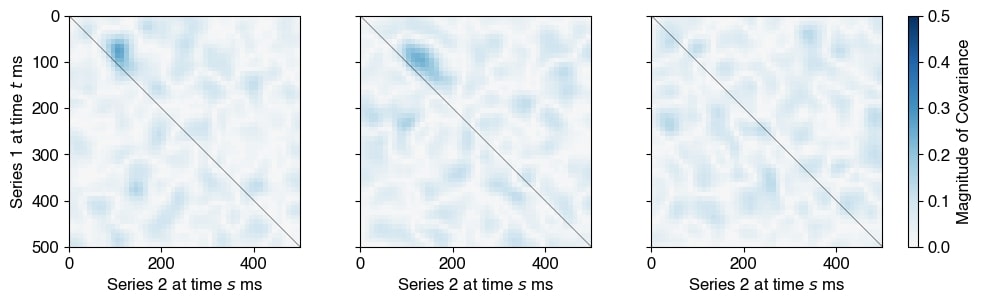

Supplement: Supplementary file 1 [file Data_Sheet_1.ZIP › images/Sighat_10_gpfa_3.jpg]

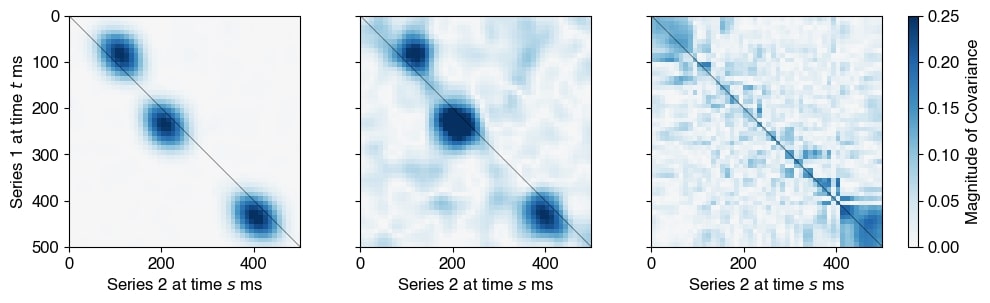

Supplement: Supplementary file 1 [file Data_Sheet_1.ZIP › images/Sighat_11_true_ladyns_dkcca.jpg]

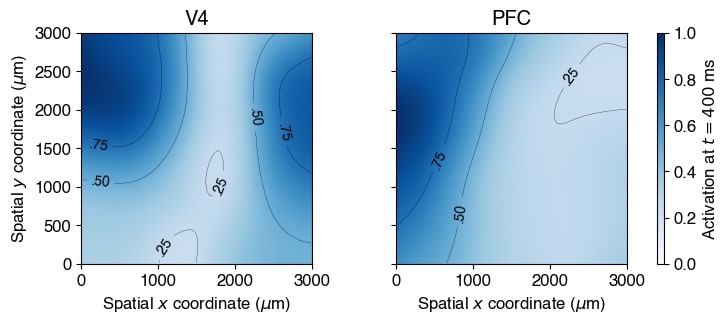

Supplement: Supplementary file 1 [file Data_Sheet_1.ZIP › images/beta_400_ct.jpg]

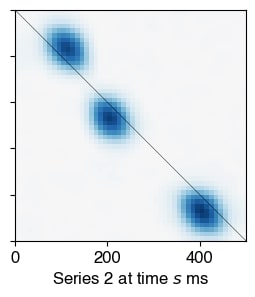

Supplement: Supplementary file 1 [file Data_Sheet_1.ZIP › images/Edspr_SSM.jpg]

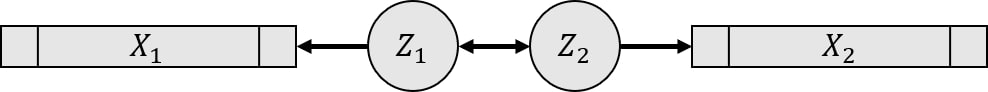

Supplement: Supplementary file 1 [file Data_Sheet_1.ZIP › images/pCCA_proposed.jpg]

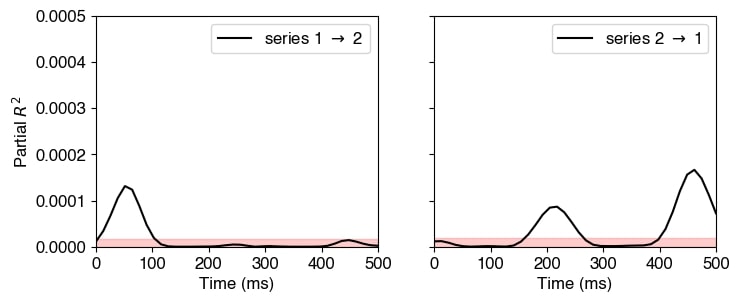

Supplement: Supplementary file 1 [file Data_Sheet_1.ZIP › images/pR2_SSM.jpg]

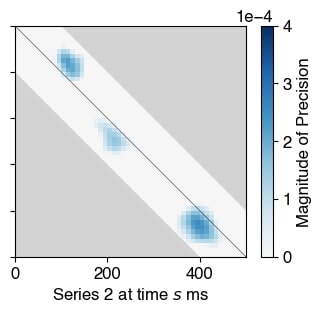

Supplement: Supplementary file 1 [file Data_Sheet_1.ZIP › images/Omega_SSM.jpg]

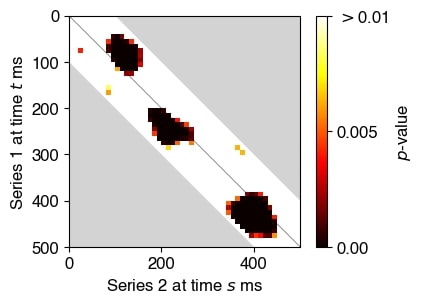

Supplement: Supplementary file 1 [file Data_Sheet_1.ZIP › images/p_SSM.jpg]

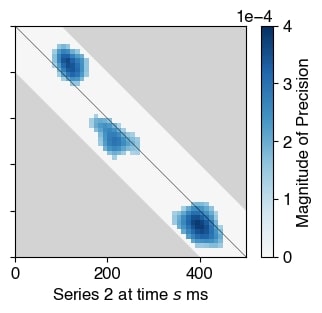

Supplement: Supplementary file 1 [file Data_Sheet_1.ZIP › images/rej_SSM.jpg]

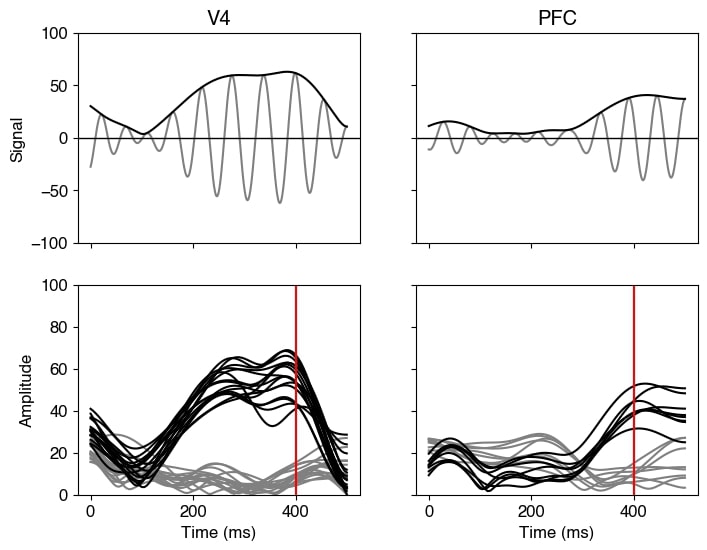

Supplement: Supplementary file 1 [file Data_Sheet_1.ZIP › images/intro.jpg]

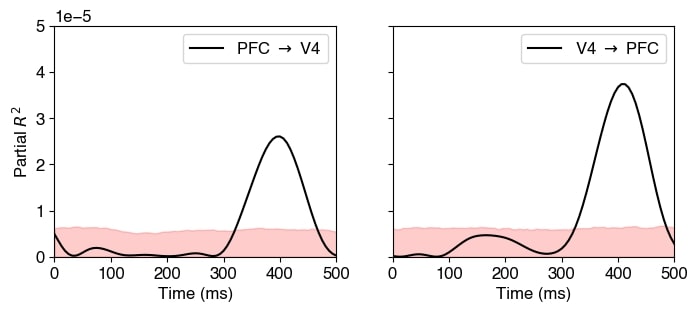

Supplement: Supplementary file 1 [file Data_Sheet_1.ZIP › images/pR2_Smith.jpg]

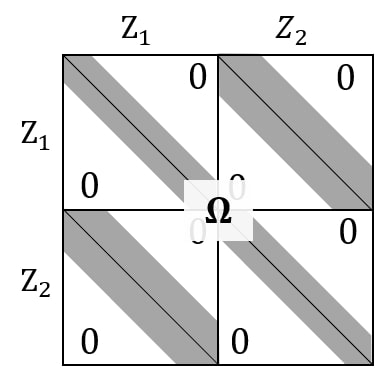

Supplement: Supplementary file 1 [file Data_Sheet_1.ZIP › images/forced_sparsity.jpg]

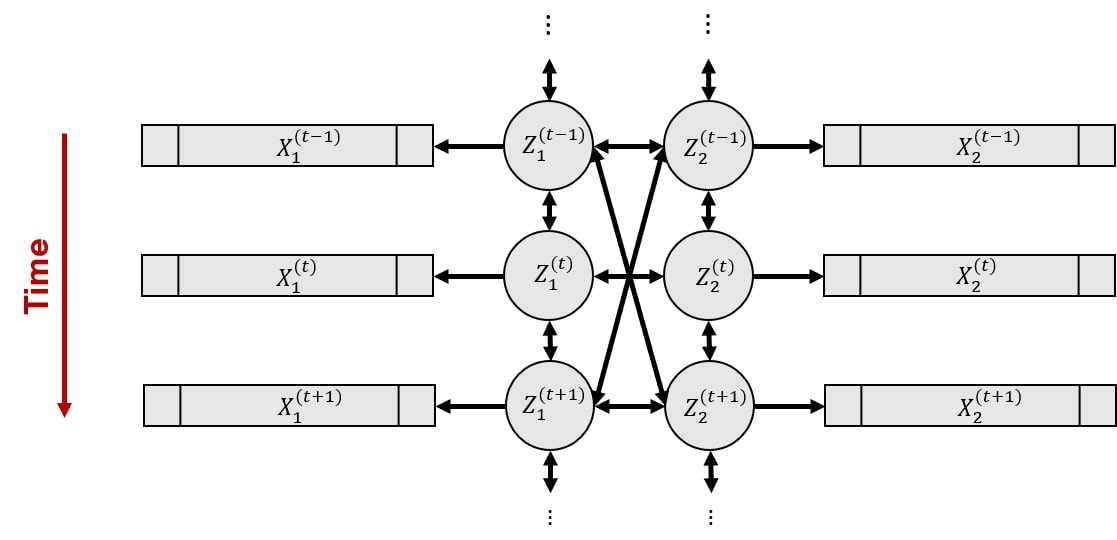

Supplement: Supplementary file 1 [file Data_Sheet_1.ZIP › images/latent_factor_model.jpg]

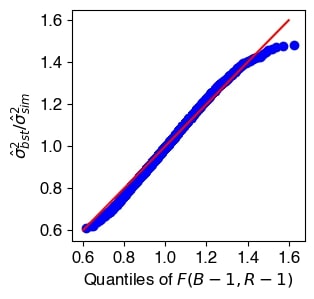

Supplement: Supplementary file 1 [file Data_Sheet_1.ZIP › images/sd_QQ_SSM.jpg]

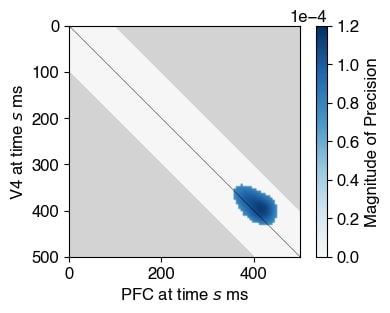

Supplement: Supplementary file 1 [file Data_Sheet_1.ZIP › images/rej_Smith.jpg]

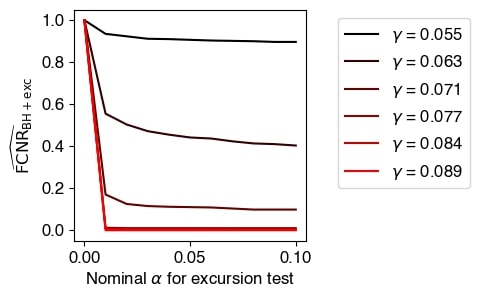

Supplement: Supplementary file 1 [file Data_Sheet_1.ZIP › images/fcnr_exc_SSM.jpg]

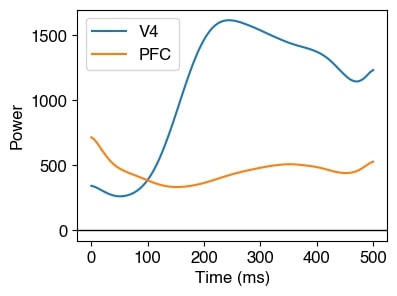

Supplement: Supplementary file 1 [file Data_Sheet_1.ZIP › images/fnorm_Smith.jpg]

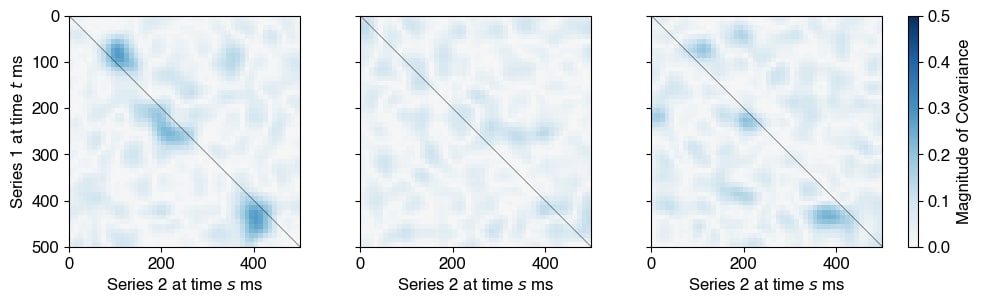

Supplement: Supplementary file 1 [file Data_Sheet_1.ZIP › images/Sighat_11_gpfa_1.jpg]

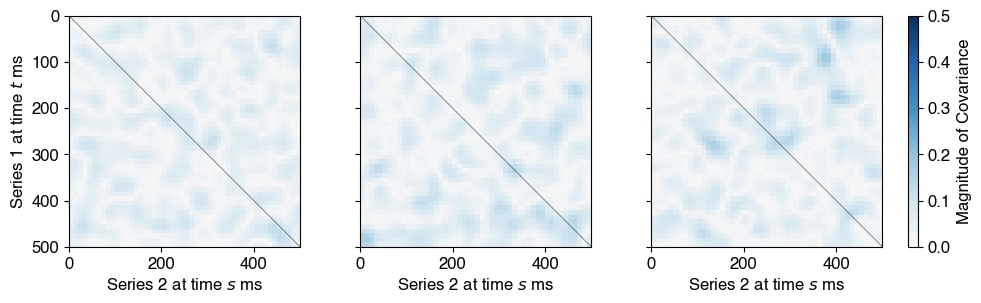

Supplement: Supplementary file 1 [file Data_Sheet_1.ZIP › images/Sighat_11_gpfa_2.jpg]

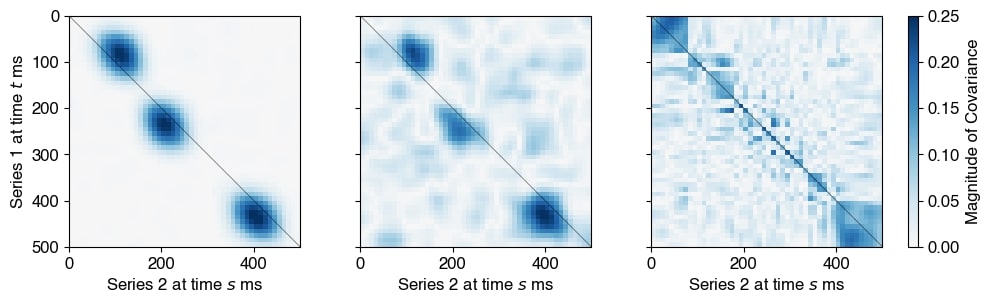

Supplement: Supplementary file 1 [file Data_Sheet_1.ZIP › images/Sighat_10_true_ladyns_dkcca.jpg]

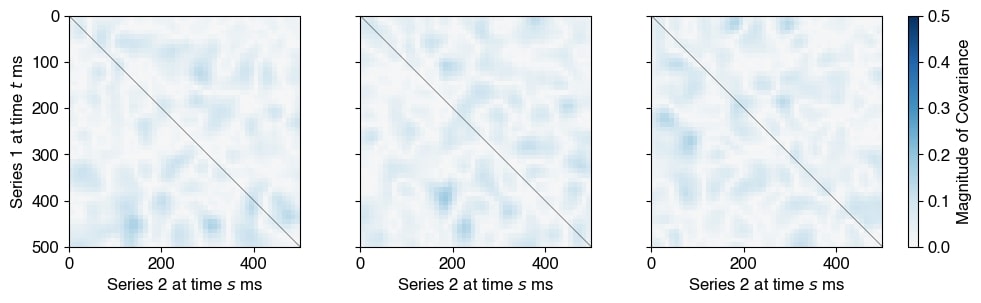

Supplement: Supplementary file 1 [file Data_Sheet_1.ZIP › images/Sighat_11_gpfa_3.jpg]

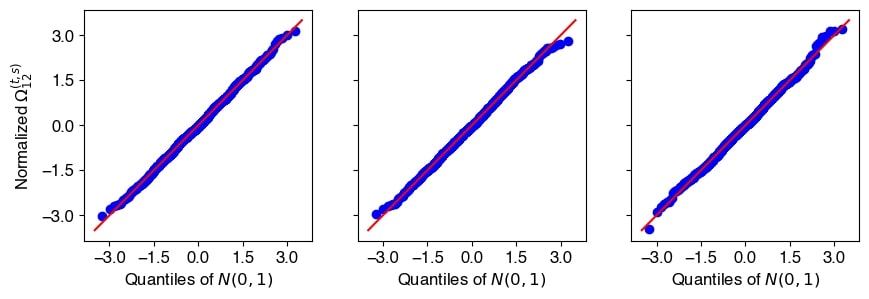

Supplement: Supplementary file 1 [file Data_Sheet_1.ZIP › images/dspr_QQ_SSM.jpg]

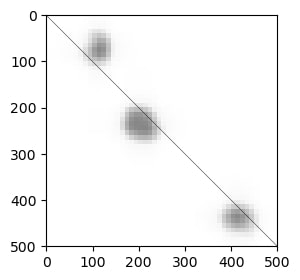

Supplement: Supplementary file 1 [file Data_Sheet_1.ZIP › images/Sighat_10_ladyns.jpg]

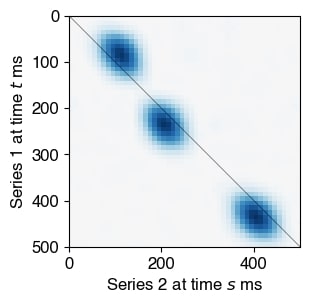

Supplement: Supplementary file 1 [file Data_Sheet_1.ZIP › images/Wtrue_SSM.jpg]

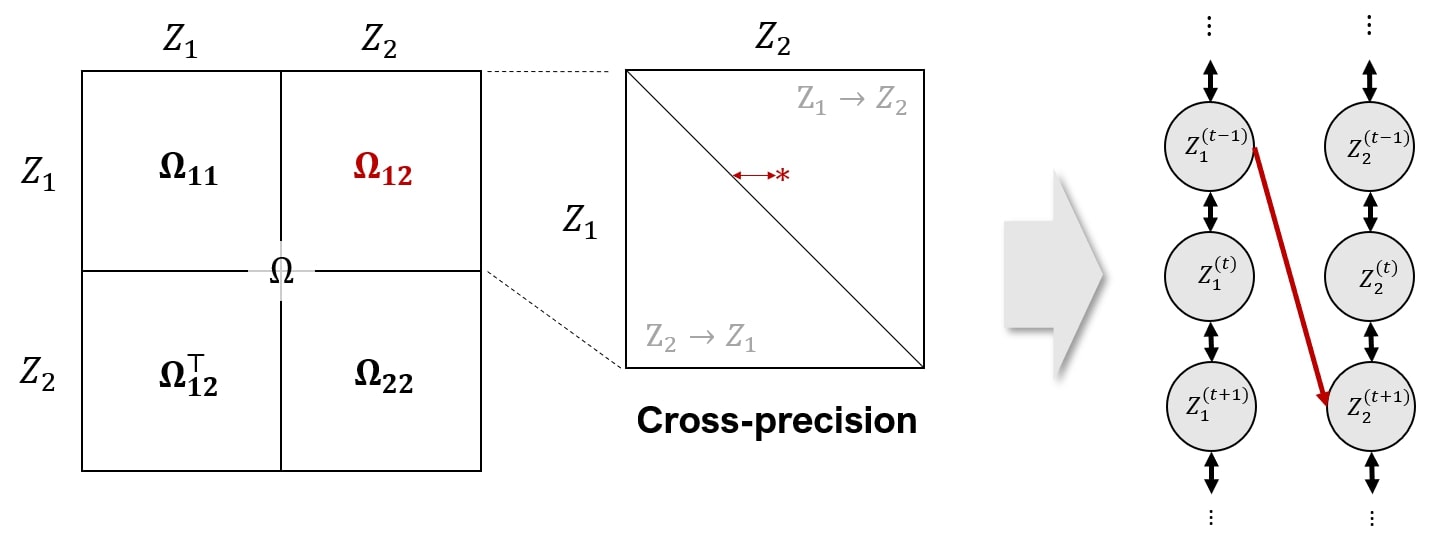

Supplement: Supplementary file 1 [file Data_Sheet_1.ZIP › images/graphical_interpretation.jpg]

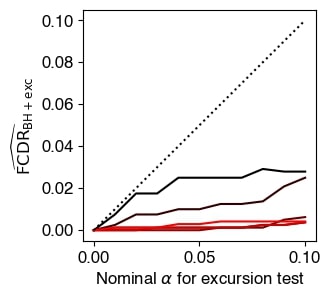

Supplement: Supplementary file 1 [file Data_Sheet_1.ZIP › images/fcdr_exc_SSM.jpg]
