# Supplementary material for: Cross-population amplitude coupling in high-dimensional oscillatory neural time series
Source: Front Comput Neurosci. 2026 Feb 3;20:1703722. doi: 10.3389/fncom.2026.1703722 (PMC12909514; doi:10.3389/fncom.2026.1703722)
Supplement: Supplementary file 2 [file Data_Sheet_2.PDF]

Supplementary Material

S1 PROOF OF THEOREMS 3.2 AND 3.3

In the model of Eqs. (5) and (6), the marginal covariance matrix S of has sub-matrices $S_{kl}^{(t,s)} = \beta_k^{(t)}\Sigma_{kl}^{(t,s)}\beta_l^{(s)\top} + \Phi_k^{(t)}\delta_{kl}^{(t,s)}$, where $\delta_{kl}^{(t,s)} = 1$ if $k = l$ and $t = s$ and 0 otherwise, for $t, s \in [T]$ and $k, l = 1, 2$. Let $u_k^{(t)} = S_{kk}^{(t,t)-\frac{1}{2}}\beta_k^{(t)}$ and $\Psi_k^{(t)} = S_{kk}^{(t,t)-\frac{1}{2}}\Phi_k^{(t)}S_{kk}^{(t,t)-\frac{1}{2}}$. We note that $u_k^{(t)\top}u_k^{(t)} = \beta_k^{(t)\top}S_{kk}^{(t,t)-1}\beta_k^{(t)} \leq 1$. Because $\beta_k^{(t)^\top}S_{kk}^{(t,t)-1}\beta_k^{(t)}$ is non-identifiable, by adjusting $\Sigma_{k,l}^{(t,s)}$ and $\Phi_k^{(t)}$ for $(k,t) \neq (l,s)$, we can assume $\beta_k^{(t)^\top}S_{kk}^{(t,t)-1}\beta_k^{(t)} = 1$. Then

$$
\begin{aligned}
u_k^{(t)\top}\Psi_{kk}^{(t,t)}u_k^{(t)} &= \beta_k^{(t)\top}S_{kk}^{(t,t)-1}\Phi_k^{(t)}S_{kk}^{(t,t)-1}\beta_k^{(t)} \\
&= \beta_k^{(t)\top}S_{kk}^{(t)-1}(S_{kk}^{(t)} - \beta_k^{(t)}\beta_k^{(t)\top})S_{kk}^{(t)-1}\beta_k^{(t)} \\
&= \beta_k^{(t)\top}S_{kk}^{(t)-1}\beta_k^{(t)} - (\beta_k^{(t)\top}S_{kk}^{(t)-1}\beta_k^{(t)})^2 \\
&= 1 - 1^2 = 0,
\end{aligned}
$$

for $t \in [T]$ and $k \in \{1, 2\}$. That is, $u_k^{(t)}$ is orthogonal to $\Psi_{kk}^{(t,t)}$.

Denoting the block diagonal matrix consisting of $\{S_{kk}^{(t,t)} : t \in [T],\ k \in \{1, 2\}\}$ by V, $R = V^{-\frac{1}{2}}SV^{-\frac{1}{2}}$ consists of sub-matrices

$$
R_{kl}^{(t,s)} = S_{kk}^{(t,t)-\frac{1}{2}}S_{kl}^{(t,s)}S_{ll}^{(s,s)-\frac{1}{2}} = u_k^{(t)}\Sigma_{kl}^{(t,s)}u_l^{(s)\top} + \Psi_k^{(t)}\delta_{kl}^{(t,s)}.
$$

Due to the orthogonality between $u_k^{(t)}$ and $\Psi_k^{(t)}$, $\det(R) = \det(\Omega)/\prod_{k,t}\mathrm{pdet}(\Psi_k^{(t)})$, and $Q = R^{-1}$ consists of sub-matrices

$$
Q_{kl}^{(t,s)} = u_k^{(t)}\Omega_{kl}^{(t,s)}u_l^{(s)\top} + \Psi_k^{(t)+}\delta_{kl}^{(t,s)},
$$

where $\Omega = \Sigma^{-1}$ is the precision matrix and $\mathrm{pdet}(A)$ and A^+ are the pseudo-determinant and Moore-Penrose pseudo-inverse of a positive semi-definite matrix A. Notice that $\Psi_k^{(t)} = I - u_k^{(t)}u_k^{(t)\top} = \Psi_k^{(t)+}$ and hence $\mathrm{pdet}(\Psi_k^{(t)}) = 1$. In turn, the negative log-likelihood under the model (Eqs. (5) and (6)) given

observed time-series $\{X_{1,[n]}, X_{2,[n]}\}_{n=1,\dots,N}$ is

$$\begin{aligned}
&\mathrm{nll}(\Sigma, \{\mu_k^{(t)}, \beta_k^{(t)}, \Phi_k^{(t)}\}_{(k,t)}; \{X_{1,[n]}, X_{2,[n]}\}_{n=1,\dots,N}) \\
&= -\log\det(\Omega) + \sum_{k,t} \log \mathrm{pdet}(\Psi_k^{(t)}) + \sum_{k,t} \log\det(S_{kk}^{(t,t)}) \\
&\quad + \mathrm{tr}(\Omega\overline{\Sigma}) + \sum_{k,t} \mathrm{tr}(\Psi_k^{(t)+} S_{kk}^{(t,t)-\frac{1}{2}} \overline{S}_{kk}^{(t,t)} S_{kk}^{(t,t)-\frac{1}{2}}) \\
&= -\log\det(\Omega) + \mathrm{tr}(\Omega\overline{\Sigma}) + \sum_{k,t} \left\{ \log\det(S_{kk}^{(t,t)}) + \mathrm{tr}((S_{kk}^{(t,t)-1} - w_k^{(t)} w_k^{(t)\top}) \overline{S}_{kk}^{(t,t)}) \right\}
\end{aligned} \tag{S1}$$

where $\overline{\Sigma}_{kl}^{(t,s)} = w_k^{(t)\top} \overline{S}_{kl}^{(t,s)} w_l^{(s)}$, $\overline{S}_{kl}^{(t,s)} = \overline{\mathbb{E}}[(X_k^{(t)} - \mu_k^{(t)})(X_l^{(s)} - \mu_l^{(s)})^\top]$ and $w_k^{(t)} = S_{kk}^{-1/2} u_k$ for $t, s \in [T]$ and $k, l \in \{1, 2\}$, and $\overline{\mathbb{E}}$ indicates the sample variance mean. Due to the first-order optimality with respect to $\mu_k^{(t)}$, $\mu_k^{(t)} = \overline{\mathbb{E}}[X_k^{(t)}]$ for $k \in \{1, 2\}$ and $t \in [T]$. On the other hand, due to the first-order optimality with respect to $S_{kk}^{(t,t)-1}$, for all $k \in \{1, 2\}$ and $t \in [T]$,

$$\nabla_{S_{kk}^{(t,t)-1}} \mathrm{nll} \quad = \quad S_{kk}^{(t,t)} - \overline{S}_{kk}^{(t,t)} = S_{kk}^{(t,t)} w_k^{(t)} \lambda_k^{(t)} w_k^{(t)\top} S_{kk}^{(t,t)},$$

where $\lambda_k^{(t)} \in \mathbb{R}$ is the Lagrange multiplier corresponding to $w_k^{(t)\top} S_{kk}^{(t,t)} w_k^{(t)} = 1$. Because $w_k^{(t)\top} S_{kk}^{(t,t)} w_k^{(t)} = 1$,

$$\begin{aligned}
1 - w_k^{(t)\top} \overline{S}_{kk}^{(t,t)} w_k^{(t)} &= w_k^{(t)\top} S_{kk}^{(t,t)} w_k^{(t)} - w_k^{(t)\top} \overline{S}_{kk}^{(t,t)} w_k^{(t)} \\
&= w_k^{(t)\top} S_{kk}^{(t,t)} w_k^{(t)} \lambda_k^{(t)} w_k^{(t)\top} S_{kk}^{(t,t)} w_k^{(t)} = \lambda_k^{(t)}.
\end{aligned}$$

Therefore, the two terms $\log\det(S_{kk}^{(t,t)})$ and $\mathrm{tr}((S_{kk}^{(t,t)-1} - w_k^{(t)} w_k^{(t)\top}) \overline{S}_{kk}^{(t,t)})$ are rewritten by

$$\begin{aligned}
\log\det(S_{kk}^{(t,t)}) &= -\log(1 - \lambda_k^{(t)}) + \log\det(\widehat{S}_{kk}^{(t,t)}), \\
\mathrm{tr}((S_{kk}^{(t,t)-1} - w_k^{(t)} w_k^{(t)\top}) \overline{S}_{kk}^{(t,t)}) &= \mathrm{tr}((S_{kk}^{(t,t)-1} - w_k^{(t)} w_k^{(t)\top})(S_{kk}^{(t,t)} - S_{kk}^{(t,t)} w_k^{(t)} \lambda_k^{(t)} w_k^{(t)\top} S_{kk}^{(t,t)})) \\
&= d_k - 1
\end{aligned}$$

Plugging it to Eq. (S1), the MLE reduces to minimizing

$$\mathrm{nll}(\Omega, w_k^{(t)}, \lambda_k^{(t)}; \{X_{1,[n]}, X_{2,[n]}\}_{n=1,\dots,N}) = -\log\det(\Omega) - \sum_{k,t} \log(1 - \lambda_k^{(t)}) + \mathrm{tr}(\Omega\overline{\Sigma})$$

such that $\mathrm{diag}(\Omega^{-1}) = \mathbf{1}$. Let $w_k^{\prime(t)} = w_k^{(t)} / \sqrt{1 - \lambda_k^{(t)}}$, $\Omega' = \mathrm{diag}(\sqrt{1 - \lambda_k^{(t)}})\ \Omega\ \mathrm{diag}(\sqrt{1 - \lambda_k^{(t)}})$, and $\overline{\Sigma}' = \overline{\mathrm{Var}}[(w_1^{\prime(1)\top} X_1^{(1)}, \dots, w_2^{\prime(T)\top} X_2^{(T)})]$. Note that $\mathrm{diag}(\overline{\Sigma}') = \mathbf{1}$. The objective is rewritten by

$$\mathrm{nll}(\Omega', w_k^{\prime(t)}; \{X_{1,[n]}, X_{2,[n]}\}_{n=1,\dots,N}) = -\log\det(\Omega') + \mathrm{tr}(\Omega'\overline{\Sigma}')$$

which is maximized when $\Omega' = \overline{\Sigma}'^{-1}$ given $w_k^{\prime(t)}$'s are fixed. sThus, the maximum likelihood estimation is equivalent to finding $w_k^{\prime(t)}$ minimizing $\log\det(\overline{\Sigma}')$ under $w_k^{\prime(t)\top} \overline{S}_{kk}^{(t,t)} w_k^{\prime(t)} = 1$ for $k \in [K]$, which is the

GENVAR procedure of Kettenring (1971). This proves the desired results for the MLE with $m_k^{(t)} = 1$. The other MLEs with $m_k^{(t)} < 1$ correspond to the non-identifiable parameter sets, which have $u_k^{(t)\top} u_k^{(t)} < 1$.

Theorem 3.2 is a corollary of Theorem 3.3. To see that, let $T = 1$ so that $X_1 \equiv X_1^{(1)}$, $X_2 \equiv X_2^{(1)}$ and $\Sigma = \begin{pmatrix} 1 & \Sigma_{12}^{(1,1)} \\ \Sigma_{12}^{(1,1)\top} & 1 \end{pmatrix} = \begin{pmatrix} 1 & \sigma_{12} \\ \sigma_{12} & 1 \end{pmatrix}$. The `GENVAR` procedure solves

$$\underset{w_1,w_2}{\operatorname{argmin}} \det \left(\overline{\mathrm{Var}} \left[w_1^\top X_1, w_2^\top X_2 \right] \right) \equiv \underset{w_1,w_2}{\operatorname{argmin}} \det \left(\begin{pmatrix} 1 & \overline{\sigma}_{12} \\ \overline{\sigma}_{12} & 1 \end{pmatrix} \right),$$

where $\overline{\sigma}_{12} = \frac{w_1^T \overline{\Sigma}_{12} w_2}{\sqrt{w_1^T \overline{\Sigma}_1 w_1} \sqrt{w_2^T \overline{\Sigma}_2 w_2}}$. This minimization problem is equivalent to the CCA problem in Eq. (1) and $\overline{\mathrm{Var}} \left[\widehat{w}_1^T X_1, \widehat{w}_2^T X_2 \right] = \begin{pmatrix} 1 & \widehat{\sigma}_{cc} \\ \widehat{\sigma}_{cc} & 1 \end{pmatrix}$, which implies $\widehat{\beta}_k = \overline{\Sigma}_{kk} \widehat{w}_k m_k$ and $m_1 m_2 \widehat{\sigma}_{12} = \widehat{\sigma}_{cc}$ for $|m_k| \leq 1$, as in Theorem 3.2.

S2 FITTING LADYNS

S2.1 Coordinate Descent Algorithm

Algorithm 1. Coordinate descent algorithm to fit LaDynS

Input:

$\{X_k : k = 1, \dots, K\}$: input data
$\Lambda \in [0, \infty]^{KT \times KT}$: sparsity penalty matrix
$\text{iter}_{\text{max}} \in \mathbb{N}_+$: maximum iteration
$\text{ths} \in \mathbb{R}_+$: threshold for convergence

Output: Ω and $w_k^{(t)}$'s which solve Eq. (10) w.r.t. Fig. 4

Initialization:

Initialize $w_k^{(t)}$ so that $w_k^{(t)\top}\overline{\text{Var}}[X_k^{(t)}]w_k^{(t)} = 1$ for all $t \in [T]$ and $k = 1, 2$. e.g.,

$$w_k^{(t)} \leftarrow \mathbf{1}/\sqrt{\mathbf{1}^\top \overline{\text{Var}}[X_k^{(t)}]\mathbf{1}}. \tag{S2}$$

and let

$$\overline{\Sigma} \leftarrow \overline{\text{Var}}[w_1^{(1)\top} X_1^{(1)}, \dots, w_2^{(T)\top} X_2^{(T)}]. \tag{S3}$$

Initialize Σ and Ω by

$$\Sigma \leftarrow \overline{\Sigma} + \lambda_{\text{diag}} I_{2T} \text{ and } \Omega \leftarrow \Sigma^{-1}. \tag{S4}$$

Iteration:

```
for iter in 1:iter_max do
    Σ_last ← Σ, Ω_last ← Ω
    Σ, Ω ← P-gLASSO(Ω_last, Σ_last, Σ̄, Λ, iter_max, ths).

    for k in 1:2 and t in 1:T do
        A ← Cov̄[X_k^(t), (w_l^(s)⊤ X_l^(s) : (l, s) ≠ (k, t))].
        b ← (Ω_kl^(t,s) : (l, s) ≠ (k, t))
        if Ab ≠ 0 then
            w_k^(t) ← Var̄(X_k^(t))^{-1} A b
            w_k^(t) ← w_k^(t) / sqrt(w_k^(t)⊤ Var̄[X_k^(t)] w_k^(t))
        end if
    end for

    Σ̄ ← Var̄[w_1^(1)⊤ X_1^(1), ..., w_2^(T)⊤ X_2^(T)].
    if max(|Σ − Σ_last|) < ths then
        break
    end if
end for
```

To update Ω, we use the P-gLASSO algorithm of Mazumder and Hastie (2012), which is more efficient than the original gLASSO algorithm of Friedman et al. (2008). The efficiency is attributed to P-gLASSO's flexibility with initial values, whereas gLASSO operates with a strict choice of initial $\widehat{\Omega}$ ($\overline{\Sigma}^{-1}$ in case of Eq. (11)). In Algorithm 1, the estimate $\widehat{\Omega}$ from the past iteration serves as a warm start for the next iteration,

so that we do not have to redo the entire paths from $\overline{\Sigma}^{-1}$ to $\widehat{\Omega}$. We can further reduce the computation cost by harnessing the banded sparse structure of Ω in Fig. 4. In Algorithm 2, we provide a modified P-gLASSO algorithm designed for the banded sparsity. The modified algorithm reduces the size of the LASSO sub-problem from $2T$ to $2d_{\text{cross}} + 2d_{\text{auto}}$ and the computational cost of a P-gLASSO iteration from $O(T^4 + T^3 N)$ to $O(T((d_{\text{cross}} + d_{\text{auto}})^3 + (d_{\text{cross}} + d_{\text{auto}})^2 N)$.

Algorithm 2. Modified `P-gLASSO` algorithm

Input:
- $\Omega_{\text{init}}, \Sigma_{\text{init}} \in \mathbb{R}^{P\times P}$: initial values, $\Sigma_{\text{init}} = (\Omega_{\text{init}})^{-1}$
- $\overline{\Sigma} \in \mathbb{R}^{P\times P}$: sample covariance matrix of a P-variate random variable
- $\Lambda \in [0,\infty]^{P\times P}$: sparsity penalty matrix
- $\text{iter}_{\text{max}} \in \mathbb{N}_+$: maximum iteration
- $\text{ths} \in \mathbb{R}_+$: threshold for convergence

Output: Ω and $\Sigma = \Omega^{-1}$ which solves Eq. (11)

Initialization:

$\Sigma \leftarrow \Sigma_{\text{init}}, \Omega \leftarrow \Omega_{\text{init}}$

Iteration:

for iter in 1:max_{iter} **do**
  $\Sigma_{\text{last}} \leftarrow \Sigma$, $\Omega_{\text{last}} \leftarrow \Omega$

  for p in 1:P **do**
    D_k : the collection of q's in $[P]$ s.t. $q \neq p$ and $\Lambda_{p,q} < \infty$
    I_p : the collection of q's in 1:P s.t. $q \neq p$ and $\Lambda_{p,q} = \infty$
    (We notate the submatrix of a matrix $A \in \mathbb{R}^{P\times P}$ of rows in $I \subset [d]$ and columns in $J \subset [P]$ by A_{IJ}. We moreover use $-p$ as a notation for $[d]\backslash\{p\}$ when it is used as a subscript of A.)

    $W = (\Omega_{-p,-p})^{-1}$ can be easily calculated by $\Sigma_{-p,-p} - \Sigma_{-p,p}\Sigma_{-p,p}/\Sigma_{p,p}$

    $\Sigma_{p,p} \leftarrow \overline{\Sigma}_{p,p} + \Lambda_{p,p}$
    $\Omega_{p,D_p}, \Omega_{D_p,p} \leftarrow \texttt{LASSO}(\Sigma_{p,p} \cdot W_{D_p,D_p}, -\overline{\Sigma}_{p,D_p}, \Lambda_{p,D_p})$ with an initial value Ω_{p,D_p}.
    $\Omega_{p,I_p}, \Omega_{I_p,p} \leftarrow \mathbf{0}$
    $\Sigma_{p,-p}, \Sigma_{-p,p} \leftarrow -W_{:,D_p}\Omega_{D_p,p}\Sigma_{p,p}$
    $\Omega_{p,p} \leftarrow (1 - \Omega_{p,D_p}\Sigma_{D_p,p})/\Sigma_{p,p}$
    $\Sigma_{-p,-p} = W + \Sigma_{-p,p}\Sigma_{-p,p}/\Sigma_{p,p}$
  end for

  if $\max(|\Sigma - \Sigma_{\text{last}}|) <$ ths **then**
    break
  end if
end for

S2.2 Sensitivity of the Algorithm to Initial Values

We tested the sensitivity of Algorithm 1 to the factor loadings $\beta_k^{(t)}$ initial values, under the simulation setting in Section 3.2. Given a simulated dataset, we ran Algorithm 1 starting from 1000 different sets of initial values for $\beta_k^{(t)} \in \mathbb{R}^{d_k}$ were randomly sampled independently across $k = 1, 2$ and $t \in [T]$ from a standard d_k-dimensional multivariate Gaussian distribution. Fig. S1 shows the LaDynS estimates $\widehat{\Omega}_{12}$ from Algorithm 1 for the same dataset and three sets of initial values. Although the three $\widehat{\Omega}_{12}$'s have different values, they yield similar inference for lead-lag relationships between the two time-series. The

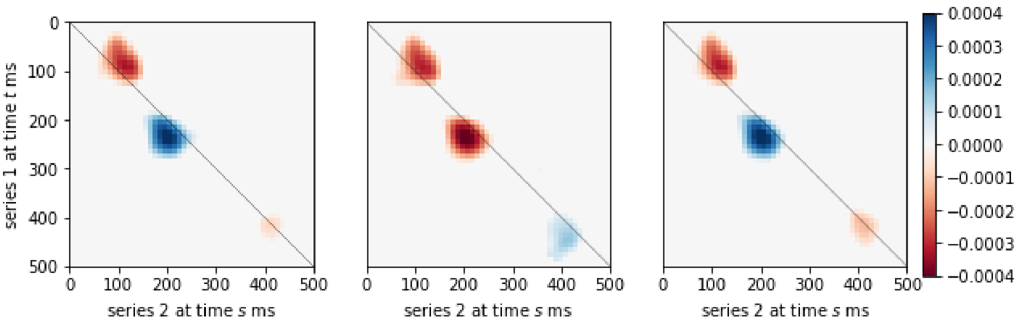

Figure S1. LaDynS estimates $\widehat{\Omega}_{12}$ by Algorithm 1 for a given simulated dataset and three sets of initial values for $\beta_k^{(t)}$'s. *The dataset was generated as described in Section 3.2, with Fig. 5(a) showing the true Ω_{12}. The three estimates $\widehat{\Omega}_{12}$ are similar and close to Ω_{12}, suggesting that Algorithm 1 is not overly sensitive to initial values.*

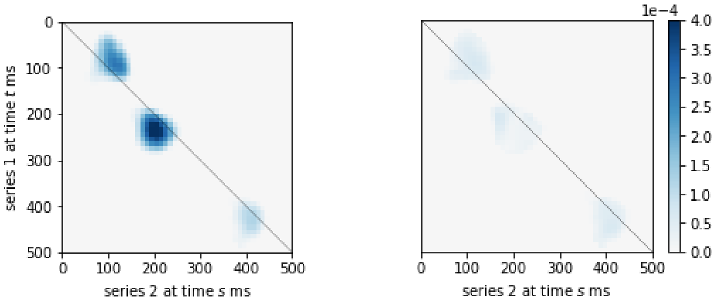

(a) **(b)**

Figure S2. (a) Mean and (b) entry-wise standard deviation of 1000 repeat Ω_{12} obtained by Algorithm 1 for a fixed dataset and 1000 different sets of initial $\beta_k^{(t)}$'s. *We used the same dataset as in Fig. S1. The mean estimated lead-lag effects in (a) are close to the true effects in Fig. 5(a), and the inferential uncertainty in (b) is small compared to the size of the effects in (a).*

most obvious differences are the signs of the estimated precision entries, but these have no implication on lead-lag inferences because the signs of the factor loadings $\beta_k^{(t)}$ and the latent precision matrix Ω are not identifiable. Fig. S2(a) confirms that the lead-lag inferences are consistent with the simulation setting in Section 3.2 and that the inferential uncertainty in Fig. S2(b) is small compared to the size of the effect in Fig. S2(a).

Next, we checked that the estimated factor loadings were similar across the 1000 runs of Algorithm 1 with different initial values. Because they are $(d_1 + d_2) \times T$-dimensional, we projected them on an arbitrary 2-dimensional subspace to visualize them. Fig. S3 shows the projected initial and final values of the factor loadings across the 1000 runs of Algorithm 1. The initial values in Fig. S3(a) are randomly distributed, by design, and converge to a distribution that is consistent across repeat simulations. Fig. S3 is consistent with our observation in Fig. S1 that the main source of the sensitivity to initial values are the signs of the precision entries in the three epochs of lead-lag relationships. Indeed, because the signs for each epoch are not identifiable, we expect $2^3 = 8$ local minima in Eq. (10), where each minimum corresponds to a set of possible signs for the three epochs. Therefore, we expect see at most 8 clusters in Fig. S3(b) (at most because clusters can overlap one another). The fitted factor loadings are not exactly equal within a cluster across repeat simulations because we optimize the likelihood Eq. (10) numerically.

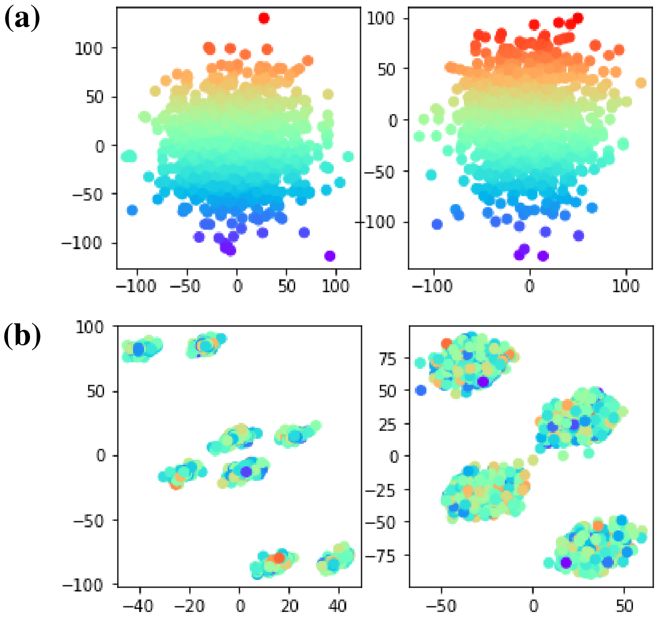

Figure S3. Scatterplots of 2D random projections of (a) 1000 different initial values of $\beta_k^{(t)}$'s and (b) corresponding fitted values after applying Algorithm 1 to the dataset used in Fig. S1. *The same projections were used for final and initial values. The left and right panels correspond to the projected β_1 and β_2, respectively. The distributions of initial values are random, by design. Fitted values converge to the same locations, further confirming that Algorithm 1 is not sensitive to initial values.*

S3 SUPPLEMENTARY RESULTS ON SIMULATED DATA

S3.1 Simulated datasets with amplitude correlation but no coherence

Here we illustrate that LaDynS can discover lead-lag relationships in amplitude where frequency-domain analyses based on coherence or phase locking cannot.

We modified the simulation setting of Section 3.2 to generate time series that have lead-lag relationships in amplitude but no coherence. We generated two sets of multi-variate LFP time series using Eq. (27), except that L_1 and L_2 were randomly phase-shifted from $L_{0,j}$:

$$L_k^{(t)} = \sum_{j=1}^{3} \beta_{kj} \cdot e^{i\omega_{kj}} \cdot L_{0,j}^{(t-\tau_{kj})} + \eta_k^{(t)}, \text{ for } k = 1, 2, \tag{S5}$$

where i in the exponential function is the complex unit, and ω_{kj} is a random phase shift sampled from Uniform distribution $U[0, 2\pi]$. One simulation dataset consisted of 1000 trials, and the coherence and amplitude correlation at $f_0 = 18$ Hz was studied. We filtered the simulated LFPs at frequency f_0 as in Section 3.2. Let $(\widetilde{X}_1^{(t)}, \widetilde{X}_2^{(t)})$ be the filtered data downsampled to 100 Hz; these data are complex-variate, of which the arguments and absolute values are the oscillatory phases and amplitudes of $L_k^{(t)}$ at frequency f_0, for $k = 1, 2$ respectively. That is, the beta power envelope $X_k^{(t)}$ is the absolute value of $\widetilde{X}_k^{(t)}$. In Section 3.2.2, we recovered the true latent factor $(Z_1^{(t)}, Z_2^{(t)})$ as

$$Z_k^{(t)} = w_k^{(t)\top} X_k^{(t)} \text{ for } t = 1, \ldots, T, \;\; k = 1, 2,$$

based on Eq. (24) and known factor loadings $(\beta_1^{(t)}, \beta_2^{(t)})$. Here we also define

$$\widetilde{Z}_k^{(t)} = w_k^{(t)\top} \widetilde{X}_k^{(t)} \text{ for } t = 1, \ldots, T, \;\; k = 1, 2.$$

The cross-coherence $C_{12}^{(t,s)}$ between $\widetilde{Z}_1^{(t)}$ and $\widetilde{Z}_2^{(s)}$ is the population coherence between $\widetilde{X}_1^{(t)}$ and $\widetilde{X}_2^{(s)}$, whereas the cross-correlation $\Sigma_{12}^{(t,s)}$ between $Z_1^{(t)}$ and $Z_2^{(s)}$ is the population amplitude correlation. Fig. S4 shows the estimates of C_{12}, Σ_{12} and the amplitude cross-precision Ω_{12} based on the average of 200 repeats, as in Section 3.2.2. We can verify that there is no cross-coherence between the two time series and that the amplitude cross-correlation is as in Section 3.2.

To verify that LaDynS can estimate the lead-lag relationships in amplitude, we applied LaDynS to a simulated beta power envelope dataset $(X_1^{(t)}, X_2^{(t)})$. Fig. S5 shows the LaDynS estimate of Ω_{12}. Although there is no coherence between the two time series, the LaDynS estimate recovers the true cross-precision in Fig. S4(c), when coherence-based frequency-domain methods cannot find a significant coherence at any pair of time points.

S3.2 Analysis of simulated datasets with known canonical correlation matrix

To further examine the properties of LaDynS and associated inference procedures, we present the results of another simulation study. One simulated dataset consisted of $N = 1000$ i.i.d. vector time-series X_1 and X_2 of dimensions $d_1 = d_2 = 25$ and durations $T = 50$, simulated from Eqs. (5) and (6). The latent time

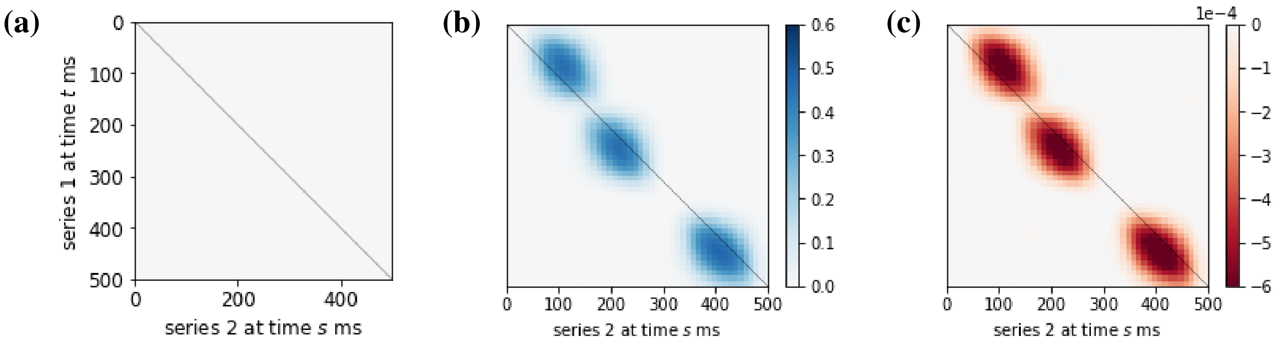

Figure S4. (a) Cross-coherence matrix C_{12}, (b) cross-covariance matrix Σ_{12} and (c) cross-precision matrix Ω_{12} of two latent time-series simulated as in Section S3.1. *Lead-lag relationships between amplitudes of the simulated time series are present, but there is no coherence between their phases.*

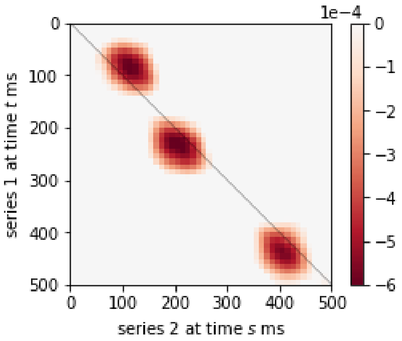

Figure S5. LaDynS cross-precision matrix estimate $\widehat{\Omega}_{12}$ to the simulated data without cross-coherence. *LaDynS can discover lead-lag relationships in amplitude where frequency-domain analyses based on coherence or phase locking cannot.*

series Z_1 and Z_2 in Eqs. (5) and (6) had zero mean vectors and covariance matrix $\Sigma = \Omega^{-1}$, with

$$\Omega = \begin{bmatrix} (\Sigma_{0,1} + \lambda I_T)^{-1} & \Omega_{12} \\ \Omega_{12}^\top & (\Sigma_{0,2} + \lambda I_T)^{-1} \end{bmatrix}, \tag{S6}$$

where Ω_{12} was the cross-precision matrix of interest. The elements of the auto-precision matrices were simulated from the squared exponential function:

$$\Sigma_{0,k}^{(t,s)} = \exp\left(-c_{0,k}(t-s)^2\right), \; k = 1, 2 \tag{S7}$$

with $c_{0,1} = 0.148$ and $c_{0,2} = 0.163$ chosen to match the LFPs autocorrelations in the experimental dataset. The diagonal regularizer λI_T was added to ensure that $\Sigma_{0,1}$ and $\Sigma_{0,2}$ were invertible, and we set $\lambda = 1$. For Ω_{12}, we considered the connectivity scenario depicted in Fig. S6(a), where the two latent times series connected in three epochs, the first with no latency, the second with series 2 preceding series 1, and the third with series 1 preceding series 2. We accordingly set the cross-precision matrix elements to

$$\Omega_{12}^{(t,s)} = \begin{cases} -r, & \text{if } (t,s) \text{ is colored red,} \\ 0, & \text{elsewhere,} \end{cases} \tag{S8}$$

where r measured the intensity of the connection. Finally, we rescaled Σ to have diagonal elements equal to one.

Once the latent time series Z_1 and Z_2 were generated, we simulated a pair of observed time series according to

$$X_k^{(t)} = Y_k^{(t)} - \beta_k^{(t)} w_k^{(t)\top} \left(Y_k^{(t)} - \mathbb{E}[Y_k^{(t)}] \right) + \beta_k^{(t)} Z_k^{(t)}, \tag{S9}$$

for $k = 1, 2$ and $t = 1, \ldots, T$, where $Y_1^{(t)}$ and $Y_2^{(t)}$ were uncorrelated baseline time series, $\beta_k^{(t)}$ were factor loadings that change smoothly over time, $w_k^{(t)}$ were canonical weights that satisfy the relationship with $\beta_k^{(t)}$ in Eq. (23), and $\mathbb{E}[Y_k^{(t)}]$ was the mean of $Y_k^{(t)}$, for $k = 1, 2$. We subtracted $\beta_k^{(t)} w_k^{(t)\top} \left(Y_k^{(t)} - \mathbb{E}[Y_k^{(t)}] \right)$ to ensure that $X_k^{(t)}$ had canonical correlation matrix Σ and the same mean as $Y_k^{(t)}$. Note that, unlike in Section 3.2, the canonical correlation matrix was known exactly. We took $Y_1^{(t)}$ and $Y_2^{(t)}$ to be the two multivariate time-series of neural recordings analyzed in Section 3.4, which we permuted to remove all cross-correlations. To reduce temporal auto-correlations, we added space-correlated white noise to the baseline time series. The amount of noise was set to be comparable to the diagonal regularization λI_T introduced in Eq. (S6). Finally we set $\beta_k \in \mathbb{R}^{d_k \times T}$ to be the factor loadings estimated in Section 3.4. The resulting latent time series $\beta_k^{(t)} Z_k^{(t)}$ and noise baseline vector $Y_k^{(t)}$ in Eq. (S9) had comparable scales and auto-correlations by construction, for $k = 1, 2$, to the experimental data in Section 3.4.

LaDynS estimation details. For this simulation, we did not need to regularize the diagonal of Ω, because the simulated time series were not smooth, and the resulting $\widehat{\Sigma}$ was invertible without the regularization. Hence we set $\lambda_{\text{diag}} = 0$. The other hyperparameters were set to $d_{\text{auto}} = d_{\text{cross}} = 10$, and $\lambda_{\text{auto}} = 0$. The penalty on the cross-correlation elements, λ_{cross}, was automatically tuned at every repeat of simulation to control false discoveries (see Section 2.3).

Results. Figure S6(c) displays the LaDynS cross precision estimate $\widehat{\Omega}_{12}$ fitted to one dataset simulated under the connectivity scenario depicted in Fig. S6(a), with connection strength $r = 0.4$ in Eq. (S8). Figure S6(d) shows the permutation bootstrap p-values for the entries of the desparsified cross-precision estimate $\widetilde{\Omega}_{12}$ (Eq. (15) with permutation bootstrap simulation size $B = 200$; see Section 2.4). Small p-values concentrate near the locations of true non-zero cross-precision entries and are otherwise scattered randomly. We applied first the BH procedure with target FDR 5% (Section 2.4) and subsequently the excursion test at the 5% significance level to all discovered clusters. The significant clusters ($p < 0.005$) are plotted in Fig. S6(e). They match approximately the true clusters in Fig. S6(a), although they exhibit random variability. To average this random variability out, we estimated Ω_{12} for each of 60 simulated datasets, and plotted their average in Fig. S6(b). The average LaDynS estimate is a close match to the true cross-precision matrix in Fig. S6(a).

Normal approximation for the p-values in Eq. (15). We investigated the validity of the Normal assumption by comparing the empirical distribution of $R = 60$ repeat estimates $\widetilde{\Omega}_{12}^{(t,s)} / \sqrt{\widehat{\mathrm{Var}}[\widetilde{\Omega}_{12}^{(t,s)}]}$ (Eq. (14)) to the standard normal distribution using QQ-plots. Fig. S7 shows QQ-plots for three randomly chosen representative time pairs (t, s) that are such that $\Omega_{12}^{(t,s)} = 0$, which validates the normal assumption. We further checked the validity of the permutation bootstrap variance estimates $\widehat{\mathrm{Var}}[\widetilde{\Omega}_{12}^{(t,s)}]$, shown in Fig. S8(b), by comparing them to the empirical variances of $R = 60$ estimates $\widetilde{\Omega}_{12}^{(t,s)}$, shown in Fig. S8(a). There is good agreement for the entries that have precision value zero, $\Omega_{12}^{(t,s)} = 0$. Fig. S8(c) further displays the Q-Q plot of the repeat ratios of permutation bootstrap over empirical estimates of $\mathrm{Var}[\Omega_{12}^{(t,s)}]$ for these

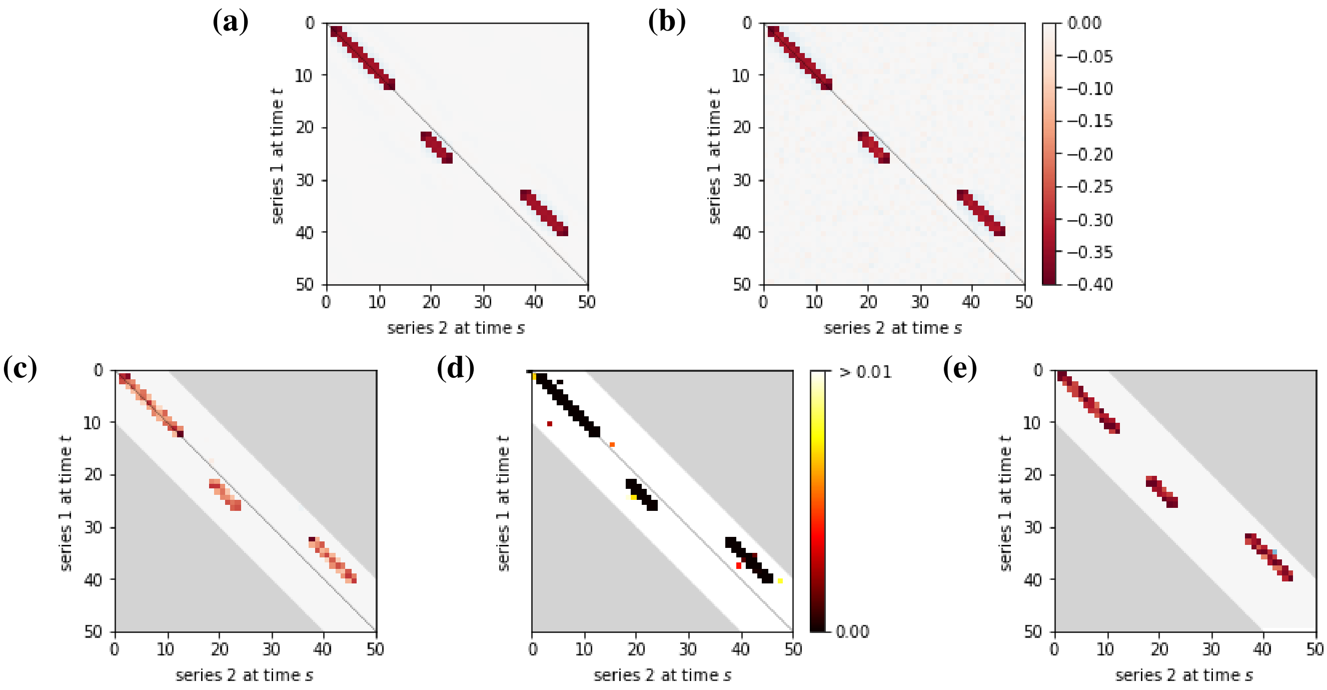

Figure S6. Output and inference of LaDynS applied to one simulated dataset from the LaDynS model. (a) *True cross-precision matrix* Ω_{12}*, for the connectivity scenario described in Section S3.2 with* $r = 0.4$*.* **(b)** *Average over* 60 *simulation datasets of LaDynS de-sparsified precision estimates* $\widetilde{\Omega}_{12}$*. There is a good match to the true* Ω_{12} *in (a).* **(c)** *Cross-precision estimate* $\widehat{\Omega}_{12}$ *for one simulated dataset. It matches (a) up to random error.* **(d)** *Permutation bootstrap p-values for the de-sparsified estimate* $\widetilde{\Omega}_{12}$*.* **(e)** *Discovered non-zero cross-precision estimates by the BH procedure at nominal FDR* 5%*. The cluster-wise p-values of the three discovered clusters by the excursion test were all smaller than* 0.5%*. All the panels but (d) share the same color bar in (b).*

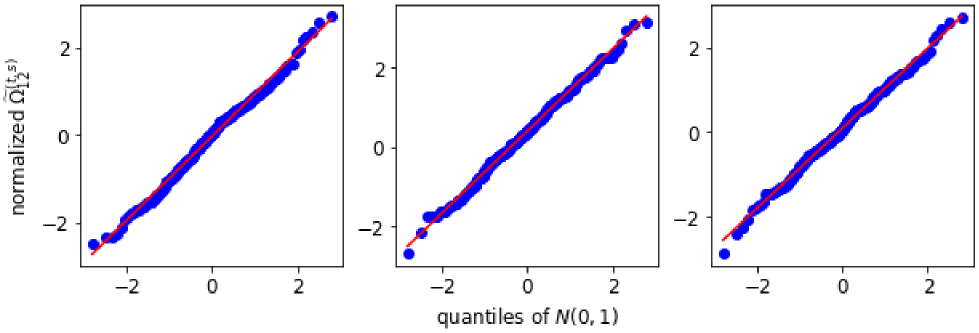

Figure S7. Null distributions of three representative entries of $\widetilde{\Omega}_{12}^{(t,s)}/\sqrt{\widehat{\mathrm{Var}}[\widetilde{\Omega}_{12}^{(t,s)}]}$ *obtained from* $R = 60$ *simulated datasets (Section S3.2), compared to the standard Gaussian distribution via QQ-plots. There is good agreement.*

entries, with $F(B-1, R-1)$ being the reference distribution. The good agreement suggests that the bootstrap estimate of $\mathrm{Var}[\Omega_{12}^{(t,s)}]$ is reliable.

FDR control. Fig. S9 shows that estimated and target FDR values match for a range of connection strengths (r in Eq. (S8)). In addition, the FNR is very low for target FDRs larger than 2%.

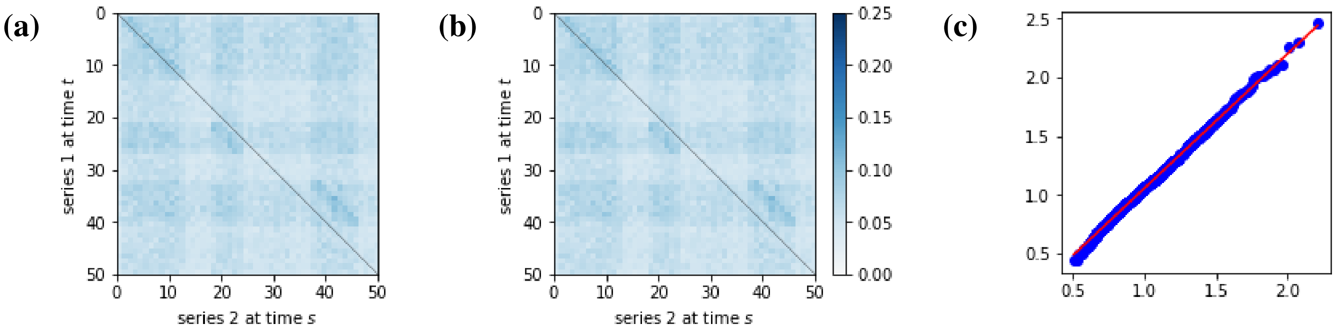

Figure S8. Standard deviations of desparsified precision elements. *Variance obtained* **(a)** *from samples from the ground-truth generative multiset pCCA model and* **(b)** *from permutation bootstrapped samples.* **(c)** *F-statistics of ratios between the two variances for null entries of* Ω_{12}*, showing good agreement.*

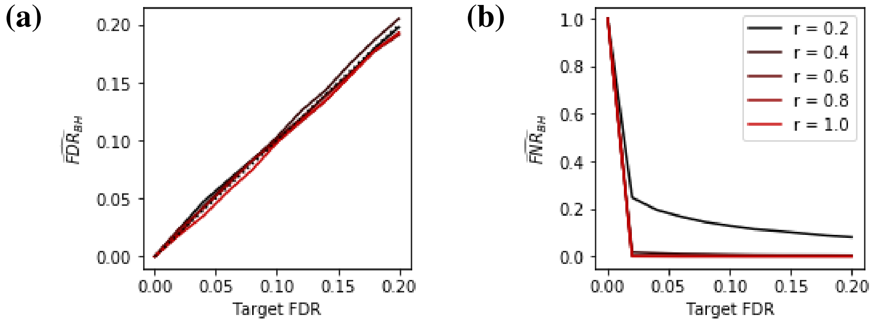

Figure S9. False Discovery Rate control for LaDynS' inference. **(a)** *Estimated false discovery rate and* **(b)** *false non-discovery rate for target FDR* $\in [0, 20]\%$*, under the connectivity scenario in Fig. S6(a), for connectivity intensities* $r = 0.2$, 0.4, 0.6, 0.8 *and* 1.0 *in Eq.* (S8)*. The dotted line is a (0,1) line.*

Excursion test. The next step was to apply the excursion test to each cluster discovered by the BH procedure at target FDR 5%. As a check on the validity of this test, Fig. S10(a) shows the quantiles of null p-values versus the theoretical quantiles of the uniform distribution on $[0, 1]$. They match perfectly so the excursion test is reliable. Finally, Fig. S10(b,c) display the cluster-wise FDR and FNR (FCDR and FCNR) for the BH procedure followed by the excursion test. The estimated FCDRs (Fig. S10(b)) are small throughout the tested range of nominal FDR values and connectivity intensities. The estimated FCNRs (Fig. S10(c)) are zero for nominal FDRs greater than 2%, which means that all connectivity epochs in Fig. S6(a) were discovered by our methods in the simulated dataset.

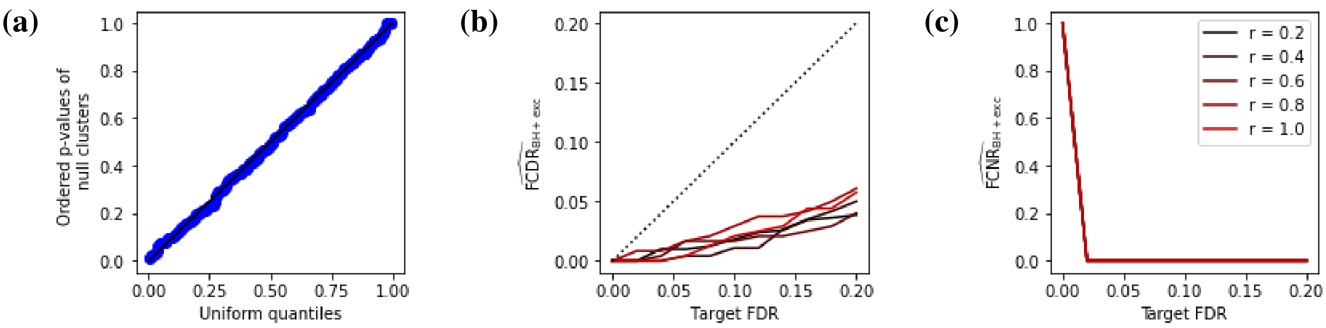

Figure S10. Performance of cluster-wise inference after excursion test. **(a)** *Q-Q plot of excursion test p-values from null clusters versus* $\mathrm{Uniform}[0,1]$ *distribution, as expected of a valid test.* **(b)** *false cluster discovery rates and* **(c)** *false cluster non-discovery rate of BH at target FDR* 10% *followed by excursion test at significance level* $\alpha \in [0, 0.10]$ *to identify non-zero partial correlations, under the connectivity scenario in Fig. 5(a), for the simulated range of connectivity intensities.*

S4 SUPPLEMENTARY FIGURES FOR THE EXPERIMENTAL DATA ANALYSIS IN SECTION 3.4

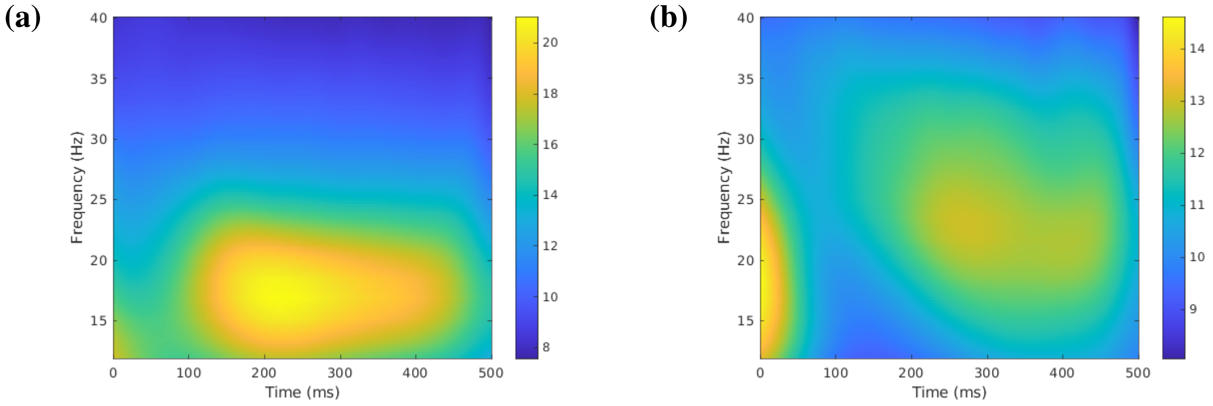

Figure S11. Averaged spectrograms across trials and electrodes in (a) V4 and (b) PFC as functions of experimental time, *cropped at frequency between* 12 *Hz and* 40 *Hz to focus on the beta band oscillations.*

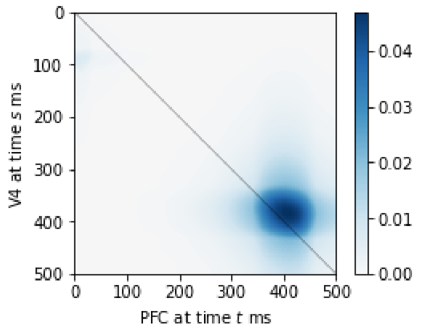

Figure S12. Latent cross-correlation matrix estimate $\widehat{\Sigma}_{12}$.

REFERENCES

Friedman, J., Hastie, T., and Tibshirani, R. (2008). Sparse inverse covariance estimation with the graphical lasso. *Biostatistics* 9, 432–441

Kettenring, J. R. (1971). Canonical analysis of several sets of variables. *Biometrika* 58, 433–451

Mazumder, R. and Hastie, T. (2012). The graphical lasso: New insights and alternatives. *Electronic journal of statistics* 6, 2125

(a)
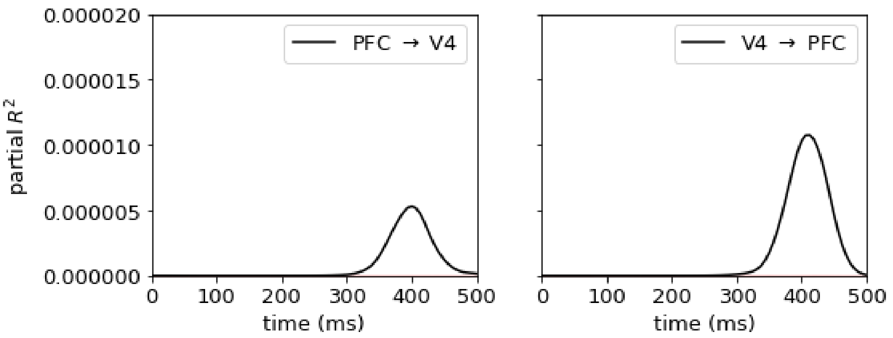

(b)
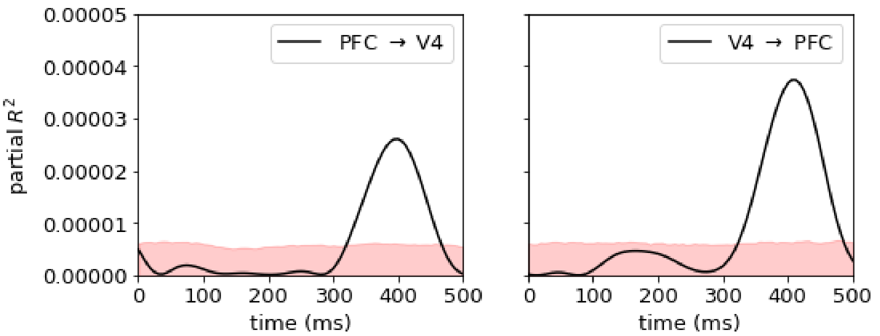

Figure S13. Estimated partial R^2 from locally stationary state-space model *based on (a) the LaDyns precision estimate $\widehat{\Omega}$ and (b) desparsified estimate $\widetilde{\Omega}$ for V4 $\to$ PFC and PFC $\to$ V4. The pink shaded areas are the 95th percentiles of null partial R^2 under independence between V4 and PFC.*

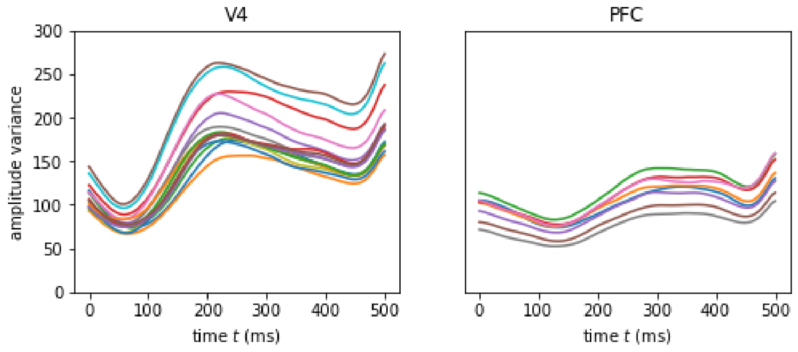

Figure S14. Estimated variances of beta amplitudes at active electrodes in V4 and PFC as functions of time. *The active electrodes were those with factor loading values larger than 75% of the maximal value at experimental time* 400 *ms (Fig. 14 shows the factor loadings at* 400 *ms over the electrode arrays). There were* 19 *active electrodes in V4 and* 16 *in PFC.*
